# Whole-body gene expression atlas of an adult metazoan

**DOI:** 10.1126/sciadv.adg0506

**Published:** 2023-06-23

**Authors:** Abbas Ghaddar, Erick Armingol, Chau Huynh, Louis Gevirtzman, Nathan E. Lewis, Robert Waterston, Eyleen J. O’Rourke

**Affiliations:** ^**1**^ Department of Biology, College of Arts and Sciences, University of Virginia, Charlottesville, VA 22903, USA.; ^2^ Bioinformatics and Systems Biology Graduate Program, University of California, San Diego, La Jolla, CA 92093, USA.; ^3^ Department of Pediatrics, University of California, San Diego, La Jolla, CA 92093, USA.; ^4^ Department of Genome Sciences, University of Washington, Seattle, WA 98195, USA.; ^5^ Department of Bioengineering, University of California, San Diego, La Jolla, CA 92093, USA.; ^6^ Department of Cell Biology, University of Virginia School of Medicine, Charlottesville, VA 22903, USA.; ^7^ Robert M. Berne Cardiovascular Research Center, School of Medicine, University of Virginia, Charlottesville, VA 22903, USA.

## Abstract

Gene activity defines cell identity, drives intercellular communication, and underlies the functioning of multicellular organisms. We present the single-cell resolution atlas of gene activity of a fertile adult metazoan: *Caenorhabditis elegans*. This compendium comprises 180 distinct cell types and 19,657 expressed genes. We predict 7541 transcription factor expression profile associations likely responsible for defining cellular identity. We predict thousands of intercellular interactions across the *C. elegans* body and the ligand-receptor pairs that mediate them, some of which we experimentally validate. We identify 172 genes that show consistent expression across cell types, are involved in basic and essential functions, and are conserved across phyla; therefore, we present them as experimentally validated housekeeping genes. We developed the WormSeq application to explore these data. In addition to the integrated gene-to-systems biology, we present genome-scale single-cell resolution testable hypotheses that we anticipate will advance our understanding of the molecular mechanisms, underlying the functioning of a multicellular organism and the perturbations that lead to its malfunction.

## Teaser

Gene atlas of adult *C. elegans* lays out common and cell-specific transcriptional programs and cell-cell communication patterns.

## INTRODUCTION

A wide range of different cell types sustains growth and reproduction in multicellular organisms. Even a simple animal such as *Caenorhabditis elegans* develops according to a selected plan, discerns food-quality, finds mates, escapes predators, learns to associate environmental cues, and survives biotic and abiotic stressors. In *C. elegans*, these functions are carried out by around 20 broadly defined cell types and more than 150 specific cell types (*1*–*3*). Underlying the morphological and functional differences between cells, are cell type–specific networks of active genes. Therefore, to unveil the molecular mechanisms underlying the functioning of multicellular organisms in physiological and pathological conditions, we need a single-cell resolution catalog of gene expression and the ability to discern genes that are common and essential from genes that define and sustain cell identity and function and genes that enable local and distal intercellular communication. This information will enable future studies to assess how perturbations (genetic, chemical, or environmental) alter gene expression at the cellular level and how these changes in turn result in phenotypes at higher levels of organization.

Recent advances in single-cell transcriptomics and *C. elegans* cell dissociation protocols (*4*, *5*) have led to single-cell gene expression profiles of *C. elegans* embryos and larvae (*1*, *3*, *4*, *6*, *7*), and a recent preprint reports the transcriptional map of sterile, mutant, adult *C. elegans* (*8*). Here, we present the expression profiles of 180 cell types identified in wild-type and fertile adult *C. elegans*. The single-cell resolution transcriptional map presented here adds several cell types that are absent in the reported studies of embryonic, larval, and sterile adult worms. For instance, we present various germ cells and cells involved in reproduction and egg laying that are specific to the adult hermaphrodite. In addition, we sequenced >150,000 cells for a single experimental condition (in three biological replicates), giving unique robustness to this dataset.

We use this catalog of adult gene expression to explore the concepts of housekeeping gene, transcription factor (TF)–mediated cellular identity, and molecular drivers of cell-cell interaction (CCI). We identified 172 genes that meet the canonical definition of a housekeeping gene and, hence, are responsible for basic cellular maintenance across cell types and possibly kingdoms. On the other hand, with 7541 predicted TF cell type associations (some of which had been experimentally validated), we begin to elucidate, at a systems level, the relationship between transcriptional programs and the identity of cells. We also predict patterns of ligand-receptor (LR) pairs that promote molecular interactions between all the cell types identified in *C. elegans*. As a result, cell type–specific cell communication signatures are proposed, some of which we experimentally validate in vivo. Last, we present a web interface to mine our dataset, wormseq.org, that together with the abundant literature and the genetic tools available to manipulate *C. elegans* will allow the community to experimentally test the hundreds of hypotheses and predictions presented in this study.

## RESULTS

### Identification of over 180 distinct *C. elegans* cell types and subtypes

**Fig. 1.**
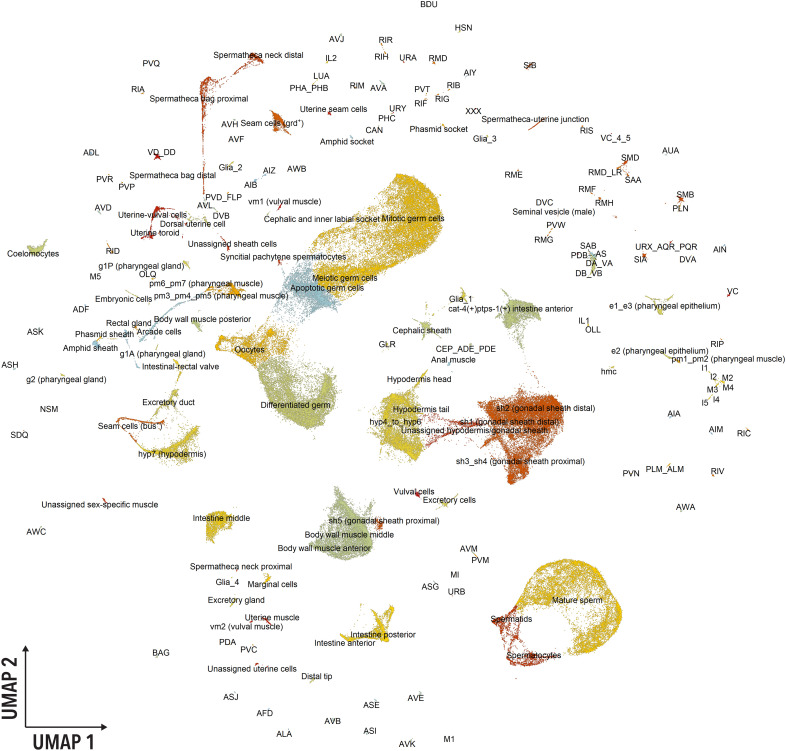
UMAP visualization of the 180 identified cell types. UMAP reduction of 154,251 cells. Each dot represents a cell. Colors indicate distinct cell types and are used to facilitate distinguishing close clusters.

Wild-type hermaphrodite *C. elegans* were harvested as young adults (YAs), defined by vulva morphology and the presence of ≤5 eggs (fig. S1A). Worms were immediately dissociated into single cells and subjected to single-cell RNA sequencing (scRNA-seq) using the 10X Chromium platform (see Materials and Methods). Three independent biological replicates were collected, and after the removal of low-quality and damaged cells (see Materials and Methods), the dataset contained 154,251 cells that passed the quality filters. The cells were then processed following the Monocle3 pipeline (*9*) and visualized using uniform manifold approximation and projection (UMAP). After Louvain clustering, the cells were separated into 170 distinct clusters ranging from 21 to 5841 cells (Fig. 1). Comparing replicates did not show batch-dependent differences in the average reads per cell (fig. S1B) or median unique genes per cell (fig. S1C). Batch-dependent differences in the proportion of cells of different types were small (fig. S1D), suggesting high reproducibility between independent experiments in the dissociation and capture of cell types. In addition, even before batch correction, cell type–specific gene expression profiles between biological replicates were highly correlated (fig. S1E; Pearson correlation coefficient: 0.86 to 0.95), which suggests that although batch differences exist, well-controlled replicates accurately recapitulate average cell types and gene expression profiles and that the effect of cell type on the measured gene expression is stronger than the batch effect.

The clusters were annotated using a multi-pronged approach that took advantage of previously published scRNA-seq data from *C. elegans* larvae (*1*, *3*) and the rich literature on *C. elegans* tissue and cell-specific markers (*10*). First, we generated a list of marker genes expressed in each cluster using Monocle3’s top_markers function (data S1). We then searched for the broad cell types in which these marker genes were expressed in the CeNGEN application (*3*) because this dataset contains scRNA-seq data from the L4 larval stage, which is the larval stage preceding the YA. This approach yielded broadly defined tissues. However, some clusters were still not confidently annotated using this approach alone because they lacked sufficient detail in the CeNGEN dataset (e.g., pharyngeal gland cells g1A versus g1P versus g2) or because the clusters were not expected to be present in CeNGEN because they are exclusive to the adult (e.g., cells involved in egg laying). We therefore used Wormbase to identify gene markers and manually annotate each unannotated cluster; the markers and rationale behind every annotation can be found in data S1.

We identified all expected broad cell types in the adult hermaphrodite *C. elegans* (*2*) including intestine, pharyngeal cell types, hypodermis, nonstriated and body wall muscle, neurons, glial cells, rectum and anus, seam, somatic gonad, vulva and uterus, excretory, coelomocytes, GLR cells, head mesodermal cells, and XXX cells. Several major cell types could be further annotated into specific cell types. The data were sufficiently exhaustive that we were able to identify cell types represented by a few or even one cell in the *C. elegans* adult. For example, we were able to identify specific pharyngeal gland cells (g1A, g1P, and g2), individual gonadal sheath cells (sh1, sh2, sh3_sh4, and sh5), and vulval muscle cells (vm1 and vm2). Last, we identified 112 of the 118 distinct neuronal classes previously reported (*11*). This includes 9 GABAergic, 51 cholinergic, 35 glutamatergic, 3 dopaminergic, 1 octopaminergic, and 2 serotonergic neuron types. Classifying the neurons by function, we found 32 sensory neurons, 22 motor neurons, 11 pharyngeal neurons, 36 interneurons, and 14 polymodal neurons. The only neurons we were not able to identify in our dataset were the I3, I6, MC, ADA, AVG, and ALN. Overall, we defined 180 specific cell types (Fig. 1 and data S1).

A limitation of current scRNA-seq technologies is that they only sequence a sample of the transcriptome of each cell. Therefore, to build more accurate transcriptional profiles for each cell type, we sought to distinguish between actual gene expression and noise. We used bootstrap resampling to estimate the average and confidence interval for the expression level of each gene per cell type (available through wormseq.org). If the lower bound of the 95% bootstrap confidence interval of the calculated scaled transcript per million (scaled TPM) was greater than 0, then we considered that gene to be robustly expressed in the analyzed cell type. Using this thresholding criterion, we estimated a median of 5871 genes robustly expressed per cell type. However, a strong positive correlation between the number of genes and number of cells per cell type indicates that the number of genes detected per cell type depends on the number of cells sequenced in each cluster (fig. S2B; Pearson correlation coefficient of 0.64). We therefore used a different metric to estimate the percentage of the transcriptome covered for each cell type given the number of cells in the cluster. The modeling (see Materials and Methods) suggests that for 177 of 180 cell types, we identified at least half of the genes expressed in that cell type. Moreover, for the majority of these cell types (135 of 180), we identified at least 75% of the transcriptome (data S2).

An unexpected consequence of our classification is the identification of transcriptionally distinct spermatheca subpopulations that had not been reported before. (Note that the rationale of all annotations can be found in data S1.) Traditionally, the spermatheca is divided into three compartments: the spermatheca neck, the spermatheca bag, and the spermatheca-uterine junction. However, our spermatheca cells clustered into five distinct clusters. Our results suggest that the spermatheca neck can be further subdivided into at least two populations of cells that relative to the uterus we name: (i) spermatheca neck distal—this cluster expresses *apx-1* and *let-502* highly (*12*, *13*) and (ii) spermatheca neck proximal—this cluster does not express *apx-1* and *let-502 *as prominently. Similarly, the spermatheca bag can be subdivided into: (i) spermatheca bag distal, this cluster prominently expresses *ajm-1* and *par-3*(*14*) and (ii) spermatheca bag proximal, which does not express these markers as prominently. On the other hand, a single cluster corresponds to the spermatheca-uterine junction. Therefore, even in an organism with every cell anatomically mapped, previously unidentified divisions of labor between cells can be uncovered using whole-body scRNA-seq.

Another unexpected observation is the presence of cell types that are not typically found in adult hermaphrodite *C. elegans*. One of these clusters expressed seminal vesicle gene markers, which are exclusively found in males. We suspect these reflect the presence of rare males (≤0.01%) in our cultures (*15*, *16*). There were also cell clusters characteristic of the L4 stage (e.g., spermatocytes and spermatids) and of early embryos. We postulate that these anachronic clusters came from younger (L4) and older adults, which are expected to rarely occur in the three independent populations of 100,000 animals that we used to isolate the cells. Once we remove the cell types corresponding to L4s, males, and embryos, we end up with 175 adult hermaphrodite cell types. It is worth noting that the fact that the dataset included the transcriptome of scarce cell types emphasizes the power of droplet-based scRNA-seq in capturing underrepresented cell populations or subtle perturbations.

### Identification of housekeeping genes

**Fig. 2.**
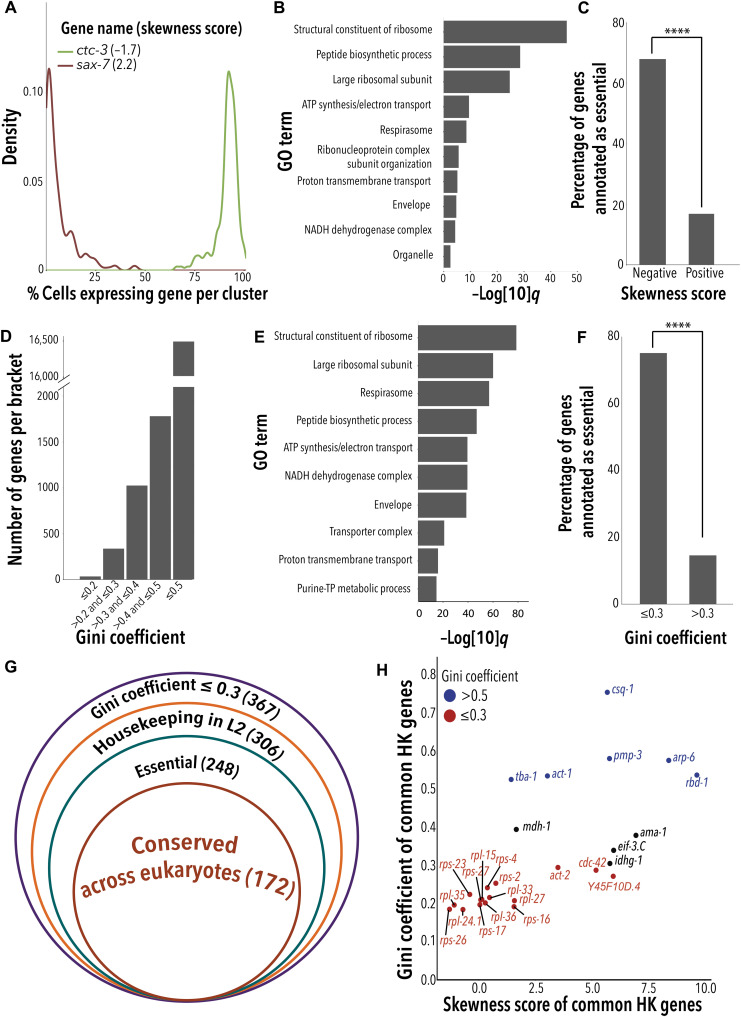
Identification of 172 experimentally validated *C. elegans* housekeeping genes. (**A**) Skewness score (in parenthesis) represents the relative abundance of mRNAs within and across cell types for a given gene. The depicted density plots illustrate two genes at the ends of the spectrum of skewness scores. (**B**) Gene Ontology (GO) enrichment analysis for genes with negative skewness scores (−log[10]*q* = −log [10] of *q* value). NADH, nicotinamide adenine dinucleotide; ATP, adenosine 5′-triphosphate; TP, triphosphate. (**C**) Proportion of essential genes (defined as lethal when knocked down by RNAi) among the 50 genes with negative and positive skewness scores. Fisher’s exact test for enrichment *P* value; ****P < 2.2 × 10^−16^, which is the smallest *P* value possible for this test. (**D**) Number of genes within each Gc bracket: ≤0.2 perfect, >0.2 to ≤0.3 good, and >0.3 to ≤0.4 adequate expression equality. Gc > 0.4 to ≤ 0.5 big and >0.5 severe expression gap. (**E**) GO enrichment analysis for genes with low Gc (<0.3) (−log[10]*q* = −log [10] of *q* value). NADH, nicotinamide adenine dinucleotide; ATP, adenosine 5′-triphosphate; TP, triphosphate. (**F**) Proportion of essential genes among genes with low (≤0.3) and high (>0.3) Gcs. Fisher’s exact test for enrichment *P* value; ****P < 2.2 × 10^−16^, which is the smallest *P* value possible for this test. (**G**) Concentric diagram to represent: Number of genes with low Gc (<0.3) in our scRNA-seq dataset, number of genes previously classified as housekeeping genes in the L2 worm (*17*), number of genes experimentally shown to be essential, and number of genes conserved across species (see also table S3). (**H**) Distribution of a set of commonly used housekeeping genes across Gcs and skewness scores. Red font indicates genes with perfect or good expression equality (Gc ≤ 0.3), and blue indicates genes with a severe expression gap (Gc > 0.5). HK, housekeeping.

Housekeeping genes can provide insight into intriguing biological questions such as which genes are under the strongest selective pressure or from a reductionist perspective, which genes are essential to cellular function in eukaryotes. Housekeeping genes also serve as references in various molecular and biochemical assays. However, it remains unclear whether commonly used housekeeping genes, or any gene, meet the commonly used criteria to define housekeepingness, namely, consistent expression across cell types and conditions, essentiality, and conservation. To assess consistent expression, we used two different criteria. We first applied a stringent criterion: abundant expression within each cell type and expression across cells. For this, we created a gene–by–cell-type matrix to define for each cell type how many cells expressed a given gene. We then used density plots to visualize the prevalence of every gene across cells within each cell type. A gene with a density plot skewed to the right (negative skewness score) is expressed in most cell types and in the majority of cells in each cell type (e.g., *ctc-3* in Fig. 2A). By contrast, a gene with a density plot skewed to the left (positive skewness score) is expressed in a few cell types, and, when expressed in a cell type, it is expressed in the minority of the cells (e.g., *sax-7* in Fig. 2A). Of the 19,657 genes we detected, only 50 genes had negative skewness scores (data S3A), indicating that, in our dataset, very few genes meet the criteria of being ubiquitously and abundantly expressed across cell types. Nevertheless, the 50 genes with negative skewness scores were enriched in “basal cellular” functions including protein translation and mitochondrial respiration (Fig. 2B), and in essential genes [identified as lethal in sub-genome (*17*) and full-genome RNA interference (RNAi) screens (*18*, *19*)] (Fig. 2C), two features in line with these genes being bona fide housekeeping genes.

It is important to acknowledge here that inherent limitations of current scRNA-seq technology could have explained, at least in part, the small number of genes robustly expressed across all cell types; however, this is unlikely to be the sole or even the main explanation (see Discussion). A more likely explanation is that we are missing housekeeping genes when using the skewness score because housekeeping genes are not necessarily abundantly expressed. Instead, housekeeping genes are expected to be consistently expressed. To identify genes consistently but not necessarily abundantly expressed, we applied a metric of inequality called the Gini coefficient (Gc) (*17*). Genes with lower Gc’s are expressed more equally across cell types, and genes with higher Gc’s are expressed in a more cell type–specific manner. In our scRNA-seq dataset, more than 90% of genes had Gc’s indicative of inconsistent expression across cell types (Gc ≥ 0.4). By contrast, 367 genes had Gc’s considered to represent good to perfect equality (<0.3) (Fig. 2D and data S3B), which suggests that they might play a role in common or core cellular functions. Gene Ontology term analysis showed that the genes with low Gc (<0.3) were enriched in basal cellular functions, including ribosomal activity and mitochondrial respiration (Fig. 2E).

Housekeeping genes are also expected to be similarly expressed across conditions. scRNA-seq is not yet available for *C. elegans* subject to perturbations (genetic, chemical, or other); therefore, we used time, in the form of a different life stage, as an alternative condition. Specifically, we looked at the overlap between genes consistently expressed (Gc < 0.3) in YAs and a list of putative housekeeping genes reported for the *C. elegans* L2 larvae (*17*). We found that all genes with high expression equality (Gc ≤ 0.2) and most genes (306 of 367) with good expression equality (Gc < 0.3) in the YAs were also consistently expressed across cell types in the L2 larvae (data S3C). Furthermore, 248 of the 306 genes were experimentally shown to be essential (lethal) in partial (*17*) and full-genome RNAi screens (*18*, *19*) (Fig. 2, F and G, and data S3D). Last, based on the conservation criteria defined by Tabach *et al.* (*20*) and as expected for essential genes, we found that most of them (172 of 248) are conserved across animals, plants, and fungi (Fig. 2G and data S3E). Therefore, the 172 gene list meets several of the ascribed but rarely tested criteria that define housekeeping genes: (i) expressed consistently across cell types, (ii) expressed consistently across conditions (e.g., developmental stages), (iii) involved in basic cellular functions, (iv) essential for life, and (v) conserved across species.

Next, we applied the Gc estimate to assess the “housekeeping-ness” of 26 genes broadly used as “housekeeping” genes for normalization of gene expression in *C. elegans* (*21*, *22*). The majority of these genes (16 of 26) had Gc ≤ 0.3, indicating that these may be appropriate reference genes (Fig. 2H). Four of the 16 genes had a negative skewness score in our dataset (*rpl-24.1*, *rpl-35*, *rps-26*, and *rps-23*), indicating that these four genes are not only consistently but also abundantly expressed across cell types (Fig. 2H). On the other hand, six commonly used “housekeeping genes”—*rbd-1*, *tba-1*, *pmp-3*, *act-1*, *arp-6*, and *csq-1*—had a Gc of more than 0.5. This severe expression gap indicates that these six genes are inadequate normalization factors (Fig. 2H); all six genes are expressed in a tissue-specific manner (fig. S3, A to F). We therefore recommend avoiding the use of *rbd-1*, *tba-1*, *pmp-3*, *act-1*, *arp-6*, and *csq-1*, especially in studies involving adult *C. elegans*. On the other hand, we here identify 172 housekeeping genes that are experimentally tested for the four criteria that have so far been axiomatically ascribed to housekeeping genes. The consistent expression of these 172 genes across cell types, conditions, and species suggest that, in line with essential functions, they are under strong selective pressure.

### Inferring transcriptional regulators underlying cell identity

**Fig. 3.**
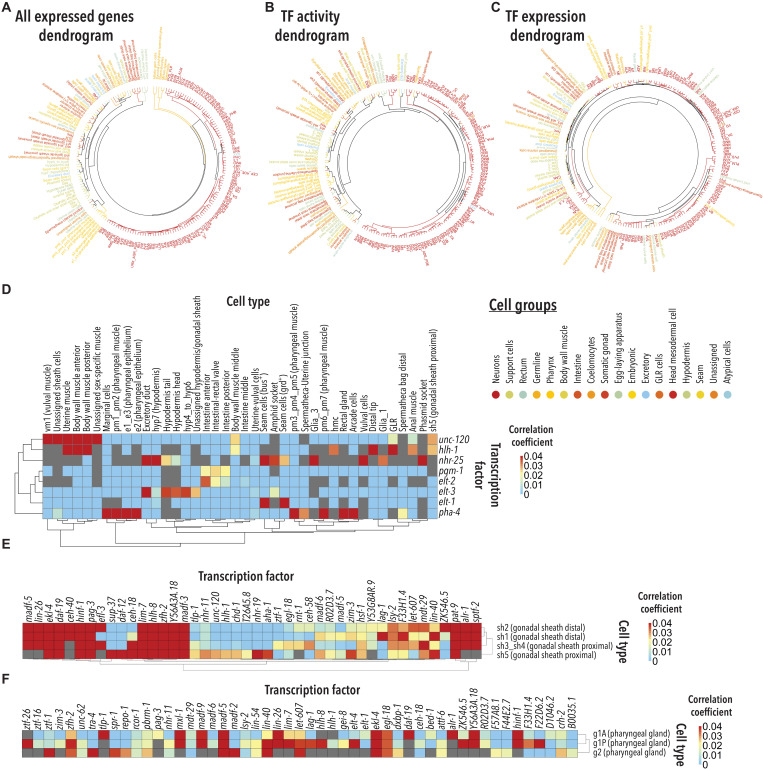
TF activity defines cell-type identity. (**A** to **C**) Circularized dendrograms depicting the relationship between cell types constructed using (A) all 19,657 genes expressed in our scRNA-seq dataset (B) predicted TF activity and (C) TF expression. Hierarchical clustering was performed using the Ward.D2 method (*74*). Subtypes of cells are colored by broadly defined cell types as depicted in the inset. (**D**) Sub-heatmap showing that the TF activity analysis recapitulates previously known TF cell type associations. (**E**) Sub-heatmap showing predicted TF activity for all gonadal sheath subtypes. Only TFs that positively correlate with at least one of the cell types are shown here. (**F**) Sub-heatmap showing predicted TF activity for all three pharyngeal gland subtypes. Only TFs that positively correlate with at least one of the cell types are shown here.

We then used an analysis successfully applied to the scRNA-seq data of the *C. elegans* L2 stage to gain insights into the regulatory programs that drive cell-specific gene expression (*6*). Briefly, the known binding patterns of TFs, as defined through chromatin immunoprecipitation sequencing (ChIP-Seq) (*23*–*25*), are correlated with the cell-specific gene expression profiles defined through scRNA-seq. To this end, we constructed regression models to predict the expression level of each gene in each of the 180 different cell types based on the strength of the ChIP-Seq peak(s) proximal to its promoter region. We then restricted correlations to TFs that were detectably expressed in our scRNA-seq dataset. This analysis yielded 7541 distinct TF cell type associations that showed correlation coefficients larger than zero (data S4 and wormseq.org). To assess the validity of these associations, which we refer to as “TF activity” associations, we tested whether the TF cell type association scores inferred from the ChIP-Seq–scRNA-seq analysis were able to predict cellular identity as defined by the expression of all 19,657 genes in our dataset. We clustered cell types using all-gene expression (Fig. 3A) and separately using TF activity scores alone (Fig. 3B). We then assessed the extent to which the “TF activity dendrogram” correlated with the “all expressed genes dendrogram.” We found that the two dendrograms were highly correlated (Baker’s gamma correlation coefficient: 0.70), demonstrating that the TF activity as defined by our model largely drives cellular identity, and, hence, it can predict cell ontological relationships between cell populations. Using only TF expression (Fig. 3C), rather than TF activity, yielded a much weaker correlation (Baker’s gamma correlation coefficient: 0.21), suggesting that using TF activity is better at inferring the TF cell type associations that drive cellular identity.

The regression analysis was able to recapitulate known TF cell type associations including *hlh-1* and *unc-120* with body wall muscle (*26*), *nhr-25* and *elt-3* with hypodermis (*27*, *28*), *elt-1* with seam cells (*29*), *elt-2* and *pqm-1* with intestine (*30*, *31*), and *pha-4 *with pharynx (*32*) (Fig. 3D). We also found that some TF cell type associations were congruent with TFs reported to act as terminal regulators of neuronal identity (*33*) (summarized in fig. S4). For instance, we found *ceh-14*, a terminal regulator of PVN identity, associated with PVN neurons. Similarly, *egl-13* was associated with BAG, URX, AQR, and PQR expression profiles. The regression analysis also suggested several previously unreported regulatory relationships (Fig. 3, D to F). For instance, *nhr-25* appears to be specifically active in the hyp7 hypodermal cells, while *elt-3* is predicted to be active in hyp4_hyp6, head, and tail hypodermal cells but not in hyp7. We also found that *unc-120* shows a high regression coefficient in several muscle cells including all sex-specific muscle cells and body wall muscle, while *hlh-1* had a high regression coefficient in anal and uterine muscle cells in addition to body wall muscles. We were also able to identify common and distinctive TFs between closely related cell types. For example, our analysis predicts that the TFs *hinf-1* and *pat-9* are common to all gonadal sheath cells (sh1, sh2, sh3_sh4, and sh5). By contrast, *lag-1* and ZK546.5 are specific to sh1 and sh2; *ztf-1* is specific to sh3_sh4; and *daf-12* and *sup-37* are specific to sh5 cells (Fig. 3E). Similarly, the TFs *madf-5*, *ekl-4*, and *egl-18* are common to all three pharyngeal gland subtypes, while *ztf-1* is specific to g1A, *daf-19* is specific to g2, and *ztf-26* is specific to g1P cells (Fig. 3F). While additional experiments will be needed to validate these inferred relationships, the results highlight the potential of combining the expression data with TF binding data to advance our understanding of the transcriptional programs responsible for driving the identity of even closely related cells.

Last, we investigated the relationship between the predicted TF activity–cell identity associations and cellular function. Specifically, we checked whether combining our scRNA-seq data with published ChIP-Seq and in vivo experimental data accurately predicted the molecular targets through which a TF of interest maintains the functional identity of a cell. For instance, the TF activity analysis predicts that the TF *dsc-1* is active in the anal muscle. In agreement with this association, RNAi against *dsc-1* causes constipation and shorter defecation cycles (*34*). Correspondingly, our scRNA-seq dataset shows 1089 DSC-1 target genes expressed in the anal muscle. Among the 1089 genes, 29 are known to contribute to normal defecation in *C. elegans*, a significant enrichment as measured by a Fisher’s exact test (*P* value = 3.183 × 10^−09^; data S5A). From the remaining DSC-1 targets, at least 70 genes are known to be required for muscle activity (*10*) and, hence, may similarly contribute to defecation (data S5B). Together, our scRNA-seq dataset, published ChIP-Seq, and published genome-wide RNAi screens allow us to postulate that DSC-1 acts cell autonomously in the anal muscle, where it controls the expression of at least 99 genes important for normal defecation (data S5, A and B). Similar to this example, our dataset enables the generation of hundreds of previously unexplored testable hypotheses across fields of study.

### Whole-body reconstruction of cell-to-cell interactions

CCIs are critical to the maintenance of the tissues and organ systems that sustain metazoan life. We previously developed the tool *cell2cell* to infer CCIs from the expression of ligand- and receptor-encoding genes across cells in single-cell transcriptomics datasets. In the original *cell2cell* study, we published a curated database of LR pairs to study CCIs in the *C. elegans* L2 larvae (*35*). Here, we use this list together with *cell2cell*’s permutation analysis (*36*, *37*) to identify CCIs between all 180 cell types and subtypes across the whole body of an adult *C. elegans* and to predict the likely molecular drivers of those interactions. A large matrix of putative interactions was obtained (see wormseq.org to browse these interactions), and below we discuss a few illustrative examples.

Without any information on CCIs and using only LR pairs and cell-specific gene expression data, *cell2cell* was able to predict known CCIs. For example, *cell2cell* predicted that the distal tip cells interact with germ cells (*38*) and that a major driver of this CCI is the molecular interaction between the ligand *lag-2* and the receptor *glp-1*; a CCI dissected through several decades of experimental studies. In addition, *cell2cell* predicted several unreported molecular interactions between cell types. For example, the LR pair *nlg-1*/*nrx-1* is the highest-scored driver of the interaction between AVA and various motor neurons. Although AVA neurons are involved in touch-induced locomotion (*39*) and *nlg-1* RNAi treated worms are resistant to touch-induced locomotion (*40*), it was not known which signaling molecules produced by AVA contributed to the touch response. However, the combination of the published data with the *cell2cell* results enables us to hypothesize that the interaction between the AVA-generated NLG-1 ligand and the NRX-1 receptor in motor neurons contributes to the touch response. Similarly, *cell2cell* predicts that *sax-7*/*pat-2* and *sax-7*/*pat-3* contribute to the interaction between DVB neurons and anal muscle cells. In support of these molecular interactions, DVB neurons innervate the anal muscle to regulate defecation, and *sax-7* mutant worms have reduced defecation rates relative to wild-type worms (*41*). However, it was not known the site of action of *sax-7* as it relates to the control of defecation or which receptor would receive its signal in the anal muscle. Together, the *cell2cell* analysis and the published work enable us to hypothesize that the molecular interaction between DVB-generated SAX-7 and the PAT-2/3 receptor in the anal muscle contributes to normal defecation in *C. elegans*. In addition, *cell2cell* predicts that the pairs *sax-7/pat-2* and *sax-7/pat-3* mediate a functional interaction between VC4 and VC5 neurons and the sex-specific muscles. This prediction is supported by the fact that *sax-7* mutants are also egg-laying defective (*41*). Therefore, the results suggest that the expression of the *sax-7* ligand in VC4 and VC5 is necessary for normal egg laying. Together, these examples illustrate the power of the *cell2cell* analysis in combination with in vivo genetic analysis to predict the molecular drivers of biologically relevant CCIs.

**Fig. 4.**
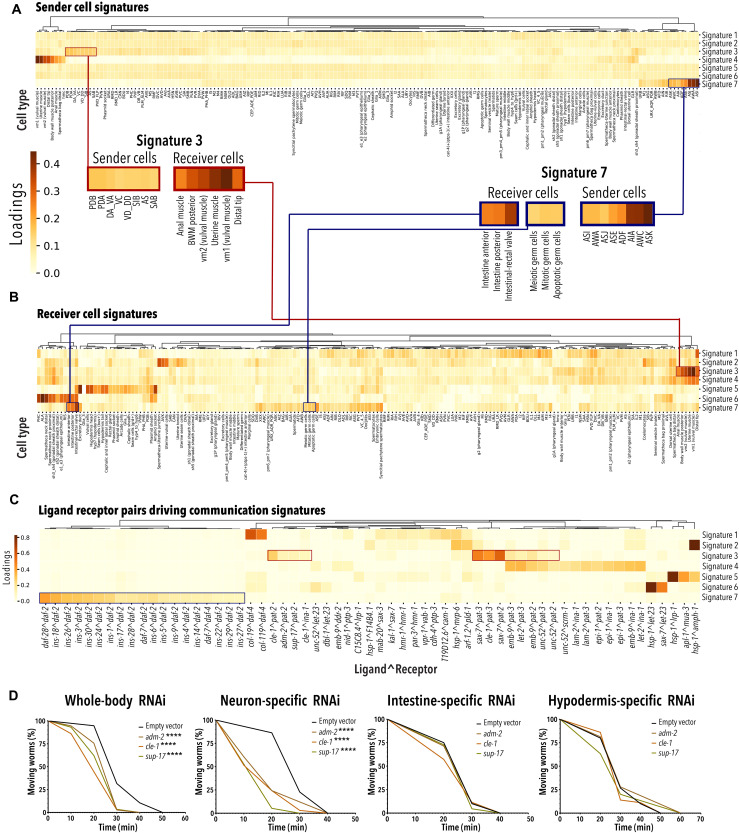
Identification of cell type–specific CCIs. (**A**) Heatmap depicts the cell types identified as the main senders across the seven communication signatures predicted by *cell2cell* in the adult *C. elegans*. Inset shows which cell types are driving the sender function in signatures 3 and 7. (**B**) Heatmap depicts cell types identified as the main receivers across the seven communication signatures predicted by *cell2cell* in the adult *C. elegans*. Inset shows which cell types are driving the receiver function in signatures 3 and 7. BWM, Body Wall Muscle. (**C**) Heatmap showing the LR pairs mediating each signature. Highlighted are the LR pairs important for signatures 3 and 7. (A) to (C) Loadings represent the importance that *Tensor-cell2cell* assigned to each element (sender or receiver cell or ligand/receptor pair) within their respective signature. (C) Only shows LR pairs that are important (loading value of >0.1) in at least one signature. (**D**) Curves depict the time it takes for RNAi-treated or control-treated (empty vector) worms to become paralyzed upon levamisole treatment (number of worms = 19 to 40; representative of two biological replicates for intestine- and hypodermis-specific RNAi and three biological replicates for WT and neuron-specific RNAi; data S7, A and B). ****P < 0.001 determined by log-rank test.

From the thousands of predictions made by *cell2cell*, we next sought to group LR pairs based on the CCIs they mediate with the goal of identifying molecular signatures that mediate the interaction between groups of cells. To do so, we used *Tensor-cell2cell*, an unsupervised machine-learning method that identifies patterns of cell-cell communication and reports them as signatures that summarize the cell types and the operative LR pairs driving their interaction (*42*). Using only our whole-body scRNA-seq data and our previously published LR pair list, *Tensor-cell2cell* identified seven unique signatures, each capturing a combination of LR pairs and groups of cell types carrying out a biological function (Fig. 4, A to C, and data S6).

Validating the *Tensor-cell2cell* approach, some of the identified signatures were well supported by previously published data. For instance, Signature 7 (Fig. 4, A to C) predicts a functional interaction between several neurons and germ and intestinal cells mediated by the insulin-signaling pathway. On the ligand side, the analysis predicts that insulin-like peptides (ILPs) are mainly produced by neurons (Fig. 4A). In support of this *Tensor-cell2cell* prediction, multiple laboratories have shown that, with a few exceptions, ILPs are generated by neurons (*42*–*46*). However, our analysis goes beyond these data because it predicts the specific neuronal subtypes that produce the ILPs in the fed adult *C. elegans*, which include ADF, AFD, AIA, AIN, ASE, ASI, ASJ, ASK, AWA, AWB, AWC, RIR, URB, and URX_AQR_PQR (Fig. 4A and fig. S5D). In addition, weaker ILP production is predicted to occur in ADL, ASG, ASH, BAG, M1, and RMH neurons. Furthermore, we can assign specific ILPs to specific neurons. For example, *ins-28* is prominently expressed in AIA, AIN, AWA, and M1 neurons, while *ins-6* is more prominently expressed in ASI and ASJ neurons (fig. S5D). On the receptor side, the most prominent receiver (receptor-producing) cells enriched in this interaction are intestinal and various germ cells (Fig. 4B). Correspondingly, several groups demonstrated the presence and functional relevance of the sole *C. elegans* insulin receptor, *daf-2*, in the germline (*47*) and intestinal cells (*48*). In addition, we find several neurons enriched as insulin-signaling receiver cells (Fig. 4B), which is supported by published work showing that insulin signaling also mediates interneuronal communication (*42*). For example, we recapitulated the insulin-signaling mediated interaction between AIA neurons and ASE neurons, which is required for salt chemotaxis learning (*49*). Specifically, in our dataset, AIA neurons prominently express *ins-1* and ASE neurons express *daf-2* (fig. S4, E and F), which are both required for salt chemotaxis learning as previously reported (*49*). The results also predict previously unexplored insulin signaling-mediated communication between other ILP-producing and receptor-producing neuronal subtypes including the *daf-2*/*daf-4*–expressing neurons (data S6).

*Tensor-cell2cell* was also able to predict previously unidentified cell communication signatures. For example, Signature 3 predicts that the ligands *adm-2*, *cle-1*, *emb-9*, *let-2*, *sax-7*, *sup-17*, and *unc-52* and their corresponding receptors *pat-2* and *pat-3* mediate the interaction between motor neurons (senders) and muscle cells (receivers). Signature 3 specifically predicts that these LR pairs mediate the interaction between the AS, DA, DB, DD, PDA, SIA, SIB, VC, and VD neurons and muscle cells from the body wall, anus, vulva, and uterus. Reassuringly, some of these predictions appear to be supported by published results. For example, CLE-1 is enriched in neuromuscular junctions (*50*), and *pat-2*, *pat-3*, *emb-9*, *let-2*, and *unc-52* contribute to muscle function (*51*, *52*). However, the site of expression and neuromuscular function of the *adm-2*, *cle-1*, and *sup-17* ligands has not been reported. We, therefore, used a levamisole-sensitivity assay to test the *Tensor-cell2cell* prediction that inactivation of these three ligands may affect neuromuscular junction function. Levamisole is an acetylcholine receptor agonist that causes continued neuronal stimulation of the muscles, leading to paralysis (*53*, *54*). Resistance or hypersensitivity to levamisole is indicative of a neuromuscular junction dysfunction (*55*). We performed whole-body and tissue-specific RNAi knockdown of the ligand genes of interest starting at the young L4 stage. When the animals reached the young adult stage, we treated them with levamisole. As predicted by Signature 3, intestine- and hypodermis-specific ligand knockdown of *adm-2*, *cle-1*, or *sup-17* did not alter sensitivity to levamisole. By contrast, whole-body and neuron-specific knockdown of all three ligands resulted in levamisole hypersensitivity (Fig. 4D and data S7, A and B). The paralysis phenotype was more pronounced in the worm strain TU3401 (Fig. 4D and data S7, A and B), which is engineered to promote RNAi knockdown specifically in the neurons of *C. elegans* (*56*). Animals treated with RNAi against the ligands showed normal chemotaxis to sodium salts, which is another reported function of the neurons expressing these ligands (PDA,VC, VD DD, and SIB) (data S7C), implying that knocking down the tested ligands does not cause pleiotropic dysfunction of the relevant neurons. Together, the match between the *Tensor-cell2cell* results and functional analyses suggests that this analysis can generate meaningful hypotheses about the molecules driving cell-to-cell communication and cell-to-cell-to-functional interactions in *C. elegans*.

### WormSeq application: Explore the whole-body transcriptional landscape of the adult *C. elegans*

To make the scRNA-seq data accessible to noncoding users, we created an RShiny application we called WormSeq. This resource is available as a web application and can be accessed using the following link: wormseq.org. WormSeq has several features, including heatmap visualization of gene expression by count and by percentage of cells expressing a gene. Users can identify cell type–specific gene markers by browsing gene marker tables or by using percentage gene expression per cell type. The interface also enables the identification of genes expressed in one cell type but not another one. Users interested in the abundance or consistency of gene expression can also browse genes by skewness score or Gc. The application also allows users to browse the regulatory program analysis data and identify TFs enriched in cell types of interest. Lastly, users can also browse the *cell2cell* analysis and identify the list of interactors driving communication between two cell types of interest.

## DISCUSSION

We present here a comprehensive single-cell atlas of a wild-type adult *C. elegans*. Although single-cell transcriptional atlases have been generated for other metazoans, including mice and humans (*57*, *58*), this scRNA-seq dataset is unique due to the following: (i) It derives from three independent populations each composed of ~100,000 animals; (ii) it was obtained from hermaphroditic, genetically homogeneous animals, which entails lower expression noise than what can be achieved in gonochoric species; and (iii) the soma, tissues, and organs of each and all adult *C. elegans* have the same cell types and number of cells (e.g., 95 body muscle cells in total). This redundancy yielded a high-resolution scRNA-seq dataset that captures all cell transcriptomes including those underrepresented in the starting worm populations (e.g., male cell types). In addition, although current scRNA-seq protocols capture only a small fraction of the total RNA molecules per cell, our oversampling of *C. elegans* cells (total of 154,251 cells) and the aggregation of cells from the same cell type enabled the reconstruction of a representative transcriptional profile for each cell type composed of a median number of 5871 genes per cell type. This level of gene activity per cell type poses interesting questions for future investigation. Which of these genes are required to maintain cell identity and function? Which ones are part of transcript reservoirs ready to act upon stress or other contexts? Which ones reflect biological or experimental noise? A more general limitation of any RNA analysis is that mRNA expression may or may not reflect protein abundance. Previous studies have shown that the correlation between mRNA levels and protein levels can be poor (*59*–*61*). Therefore, incorporating proteomic data, and in the future single-cell proteomic data, is anticipated to increase the accuracy of functional predictions.

Despite the technical limitations, the *C. elegans* transcriptional atlas reported here is composed of the gene expression profiles of 180 distinct cell types. In addition to identifying most known *C. elegans* cell types, our annotation revealed differences in expression profiles between cell types previously assumed to be the same due to morphological similarity. For example, *apx-1* and *let-502* being highly expressed in the distal but not in the proximal spermatheca neck together with the reports showing that whole-body RNAi against *apx-1* (*62*) and *let-502* (*63*) lead to dysregulated expansion of the germ line, suggest that the distal spermatheca neck cells may contribute to tumorous processes in the germ line. Therefore, the transcriptome-based annotation of *C. elegans* cells presented here opens doors to learn previously unexplored cell-specific biology.

In this study, we also use the single-cell data to begin to address three fundamental questions about the relationship between gene expression and cellular function: Which, if any, genes meet the definition of housekeeping gene? What transcriptional programs generate and maintain cellular identity in *C. elegans*? Which genes mediate the interactions between cells in this metazoan?

### What genes are housekeeping?

Using scRNA-seq we were able to directly test for a feature commonly attributed to housekeeping genes: consistent expression across cell types. We scored all genes in our dataset based on the abundance and consistency of expression across cell types using a skewness score or only for consistency of expression using a Gc. Although skewness score and Gc positively correlate with each other (Pearson correlation coefficient = 0.66), the Gc is more likely to have fewer false negatives because housekeeping genes are not necessarily abundantly expressed. Supporting the use of the more permissive Gc to identify housekeeping genes, the resulting list of consistently expressed genes (Gc ≤ 0.3) is enriched in genes essential to *C. elegans* survival (276 of 367) to an extent similar to the most restrictive skewness set (34 of 50). Furthermore, 248 of the 276 genes are consistently expressed in two very distinct ontogenetic stages, the L2 larvae and the adult *C. elegans*, showing that the Gc analysis applied to one condition can capture genes consistently expressed across conditions.

Because our housekeeping genes analysis is limited to the N2 wild-type *C. elegans* strain, and it compares only two developmental stages (L2 larvae and young adult), it may be too inclusive. Additional scRNA-seq experiments in other genetic backgrounds, developmental stages, or in the presence of abiotic or biotic stressors may further narrow the number of housekeeping genes or even challenge the concept of housekeeping gene altogether. On the other hand, scRNA-seq limitations such as low depth of sequencing and the incomplete sampling of each cell could account for false negatives. Nonetheless, the 248 genes found in the Gini analysis represent a solid beginning. As expected, these 248 genes are enriched in basic cellular functions including mitochondrial function, protein synthesis (e.g., ribosome and protein translation), and protein stability (e.g., chaperones). Intriguingly, we did not find DNA synthesis/replication genes in this set, likely a reflection of the fact that apart from the germ line, cells in the adult *C. elegans* are post-mitotic. We also did not find enrichment for RNA synthesis genes, although at least some of these genes are well represented across cell types (e.g., the RNApol encoding gene *ama-1*). Nevertheless, of the 248 genes consistently expressed in *C. elegans*, we found that 172 are conserved (*20*) in organisms ranging from yeast to rice and humans (data S3E), and hence, they could be part of the core of genes indispensable to build and maintain a eukaryotic cell.

### What transcriptional programs generate and maintain cellular identity in *C. elegans*?

Our regression analysis of ChIP-Seq data with the cell type–specific gene expression profiles revealed 7541 TF-cell type expression profile associations. Some of the TF-cell type associations were known, and, consequently, they validate the approach. However, we also generated predictions that, in line with the high resolution of our dataset, reveal TF-cell type associations that are distinct even between closely related cell subtypes such as pharyngeal gland g1A and g1P. In WormSeq, the web interface accompanying this study, users can search for all the TFs predicted to be active in each cell type and all the cell types in which a given TF is predicted to be active. As the number of TFs with ChIP-Seq data increases from the 362 TFs used here to encompass the 900 or so TFs predicted to exist in *C. elegans* (*64*), the association between TF binding patterns and cell-specific transcriptional profiles should become even more powerful in revealing regulatory relationships. Also, while we only evaluated the activating role of TFs, other advances may permit the investigation of negative regulators. Last, unlike our transcriptomic data, the ChIP-Seq data we relied on were generated in bulk samples; this may lead to inaccurate results for TFs that are only active in a small set of cells. Therefore, as single-cell ChIP-Seq becomes more popular, we expect this kind of analysis to become more accurate for more sparsely expressed TFs.

By combining our scRNA-seq with published ChIP-Seq and functional data, we proposed cell-type TF targets triads that may mediate cellular function and morphology. For example, knockdown of the gene encoding the TF *dsc-1* and of several of its ChIP-Seq targets can alter defecation cycles in the worm (*34*). Our analysis predicts that *dsc-1* is active in anal muscle and that 29 of its downstream targets involved in defecation are expressed in the anal muscle. Therefore, we hypothesize that DSC-1, its 29 downstream effectors, and likely another set of 70 target genes that yield more general muscle phenotypes but are expressed in the anal muscle orchestrate a cell-autonomous transcriptional program critical for normal defecation in *C. elegans*. The results presented in this study promise to accelerate advances by reducing the number of candidate genes for functional studies and by placing molecular players in their anatomical sites of action.

### Which genes mediate the interactions between cells in *C. elegans*?

We used the algorithm *cell2cell* and a curated list of LR pairs to make thousands of predictions about the gene pairs mediating the interactions between the cells of the adult *C. elegans*. We also used a permutation analysis and *Tensor-cell2cell* (*65*) to identify cell type–specific communication signatures. Validating our approach, we detected known CCI-LR associations including insulin signaling-mediated neurons–germ line and neurons–intestine communication. In addition, even for this well-characterized communication pathway, our analyses provided previously unexplored testable hypotheses including the specific neurons that produce the insulin-like ligands in the adult worm.

Our predictions, however, are only based on the expression of LR pairs without accounting for spatial constraints. This omission may lead to predictions that do not match the biology, most notably membrane-bound LR pairs predicted to mediate the interaction between physically distant cells. For this reason, in our web interface WormSeq, we included a feature that allows users to browse our CCI-LR predictions by LR class: (i) membrane-bound, (ii) ECM component, and/or (iii) secreted. We recommend potential users to use our CCI analysis in combination with *C. elegans* anatomy databases (e.g., wormatlas) to determine the relevance of the predictions. In addition, we were unable to explore the spatial properties of CCIs as we had previously done (*35*) due to the absence of an adult-specific three-dimensional (3D) digital atlas similar to the one available for L1 worms (*66*). Last, the analysis is limited to present LR interaction data. As more accurate and extensive LR interaction data become available, we expect this analysis to lead to a more comprehensive map of the molecules mediating the interactions between cells.

Overall, this study is a major step forward toward identifying the key molecular players, whose function or dysfunction defines the functional status of the intracellular and intercellular gene networks that constitute an animal. Putting together, (i) genetic tractability, (ii) stereotypical number and physical interactions between cells dissected to the level of synapses, (iii) known lineage for every cell in the body, and (iv) existing scRNA-seq datasets of the wild-type embryo, two larval stages, and now the adult will enable the development of tools that track gene expression across space and time for a whole living animal, as well as predictive models of cellular, tissue, organ, and ultimately whole-animal function. These tools can help many fields in at least two ways: (i) They may tell us how much information and of which kind is necessary to develop in silico models that can accurately predict the effect of perturbations (biological, chemical, physical, or others), and (ii) the tools themselves may serve as the basis or guide the development of predictive tools for more complex organisms.

## MATERIALS AND METHODS

### *C. elegans* strains and husbandry

*C. elegans* N2 (Bristol, UK), MGH171 (sid-1(qt9);alxIs9[vha-6p::sid-1::SL2::GFP]), JM43 (rde-1(ne219);xkIs99[wrt-2p::rde-1::unc-54 3’UTR]), and TU3401 (sid-1(pk3321);uIs69 [pCFJ90 (myo-2p::mCherry) + unc-119p::sid-1] were obtained from the Caenorhabditis Genetics Center (CGC). All strains were typically grown at 20°C on Nematode Growth Media (NGM) plates seeded with *Escherichia coli* strain OP50. Bacterial strains used for RNAi were obtained from the Ahringer library (*67*).

### Sample preparation and scRNA-seq

A synchronous population of L1 worms was obtained by double bleaching gravid N2 *C. elegans* with hypochlorite followed by four washes in S-buffer. The released eggs were then allowed to hatch in the absence of food in S-buffer over a period of 18 hours. Approximately 100,000 synchronized L1 worms were then grown in NGM plates seeded with HT115 bacteria at 20°C for approximately 55 hours. At 55 hours after seeding, worms were staged under a microscope to ensure that the bulk of the population had reached the young adult stage. Young adult worms were then harvested in S-buffer and centrifuged at 1300*g* for 1 min. The worm pellet was washed until the suspension was no longer turbid (two to three times) and then transferred to a 1.5-ml Eppendorf tube. The cuticle was then disrupted by incubating the worms in 200 μl of SDS–dithiothreitol (DTT) [20 mM Hepes (pH 8.0), 0.25% SDS, 200 mM DTT, and 3% sucrose] (*68*) for 4 min. Immediately after SDS-DTT treatment, 800 ml of egg buffer was added to the treated worms, the worms were centrifuged, the supernatant was aspirated, and the worm pellet was washed five times in egg buffer (118 mM NaCl, 48 mM KCl, 2 mM CaCl_2_, 2 mM MgCl_2_, and 25 mM Hepes at osmolarity of 340 Osm). After the final wash, egg buffer was added to a final volume of 1 ml and the worm solution was then transferred to a 15-ml conical tube. A total of 500 μl of Pronase (350 U/ml; EMD Millipore Corp.) was added, and the worms were then dissociated into single cells by passing them through a 21-gauge needle about 20 times. The worm/cell lysate was centrifuged at 4°C for 1 min at 200*g*, and then most of the supernatant, containing dissociated cells, was transferred to a new 15-ml conical tube, leaving behind enough liquid for a second round of dissociation. After passing the worm lysate through the needle for a second time, the samples were centrifuged (4°C for 1 min at 200*g*), and then the supernatant was transferred to the same tube containing the cells from the first transfer. The cells were then centrifuged at 4°C for 5 min at 500*g*, and the cell pellet was washed three times in egg buffer containing 1% bovine serum albumin gently pipetting the cells with wide-end tips. Last, to separate single cells from bigger chunks of tissue, the cell suspension was gently passed through a 10-μm filter.

For single-cell capture, approximately 15,000 *C. elegans* cells were mixed with the reverse transcriptase solution and then loaded onto each channel of the 10X Chromium Controller (we used a total of 10 channels for three biological replicates). The libraries were then built following the Chromium Next GEM Single Cell Kits v3.1 published protocols and then sequenced on an Illumina NextSeq 500 platform. Note that, with this methodology, we are only capturing polyadenylated transcripts, therefore excluding most noncoding RNA. In addition, this method does not allow for capturing alternative splicing variants.

### scRNA-seq data processing

The scRNA-seq data was first processed following the CellRanger pipeline. Reads were mapped to a modified version of the WormBase WS260 reference transcriptome that had transcript 3′ untranslated regions extended by 0 to 500 base pairs (*1*). To distinguish cells from empty droplets, we used the knee plots reported by CellRanger to set a unique molecular identifier (UMI) threshold below which droplets were considered empty. The expression matrix generated by CellRanger was then decontaminated for ambient RNA using DecontX (*69*). We then followed the Monocle3 pipeline to perform dimensionality reduction and clustering (*9*). First, we combined all three biological replicates into a single cds object. We then used Monocle3’s preprocess_cds function (method = “PCA,” num_dim = 200), which normalizes the data by log factor and generates a lower dimensional space for downstream dimensionality reduction. Next, we used Monocle3’s align_cds function to perform further background correction and remove unwanted batch effects, which we noticed came mostly from the different samples. We then performed UMAP dimensionality reduction on the matrix using Monocle3’s reduce_dimension function run with default parameters. Last, we used Monocle3’s cluster_cells function to define individual clusters of cells using the Louvain algorithm (*k* = 50).

After clustering, we noticed that there were clusters containing mostly cells with a high mitochondrial fraction (mitochondrial-only UMI/total UMI > 0.2). These clusters were removed because high mitochondrial fraction is an indication of damaged cells (*70*). We then reperformed dimensionality reduction and clustering on the remaining cells as described above. We also noted that some cells labeled “intestine middle” prominently expressed hypodermal gene markers and were therefore removed from the data because they were likely intestine-hypodermis doublets.

### Cell type annotation

To annotate the different clusters of cells with their corresponding cell types, we used Monocle3’s top_markers function to identify for every cluster a list of 10 gene markers. We then used the CeNGEN application (*3*) to broadly define where these genes are typically expressed in the L4 worm. In addition to the L4 data, we used gene markers identified through scRNA-seq of L2 worms (*1*). The annotation of the L2 worms was more detailed than the CeNGEN data and allowed us to annotate several clusters more carefully. We also used Wormbase to identify gene markers for cell types that were absent from the L2 and L4 data and those that could not be confidently annotated using the L2 and L4 data alone. Last, we found that certain clusters contained several distinct cell types. We created subsets of these clusters on which we performed dimensionality reduction and clustering to differentiate these cell types. This allowed us to improve our annotation and distinguish between cell types, which were previously bundled together. We must note however that even after subclustering, certain cell types could still not be distinguished (e.g., URX_AQR_PQR). A detailed rationale for the annotation can be found in table S1.

### Computing gene expression by cell type

The aggregate gene expression profile for a cell type was computed following the method described by Packer *et al.* (*1*). We first used Monocle3’s normalized_counts function to normalize the gene by cell expression matrix by size factor alone. For every cell type, we then (i) subsetted the gene by cell matrix to include only those cells belonging to that cell type, (ii) took the mean of each row (gene), (iii) calculated the sum of the values obtained in (ii), (iv) divided each value in (ii) by the sum calculated in (iii), and last (v) multiplied all values by a million to obtain a scaled TPM.

We also performed the same calculations using bootstrap resampling to estimate a confidence interval for the expression of each gene by cell type (*1*). For every cell type with N number of cells, we randomly sampled with replacement N cells from that cell type and calculated gene expression as described above. This was performed 1000 times for each cell type, and we then used the resulting distribution of scaled TPMs to compute confidence intervals (95 and 80%).

### Estimating transcriptome coverage of every cell type

To estimate the fraction of the transcriptome covered for every cell type in our dataset, we used the method described by Taylor *et al.* (*3*). We performed 100 iterations of down-sampling for each cell type calculating scaled TPM as described above with each iteration. We then plotted the number of genes by the number of cells for every cell type (fig. S2C). On the basis of the shape of the curves (fig. S2C), we modeled the relationship between gene number and cell number using a three-parameter log-logistic function (*71*). Using that model, we calculated a predicted maximum number of genes per cell type (*G*_MAX_) and a predicted number of cells that would allow us to identify half of *G*_MAX_ for every cell type (data S2). This allowed us to generate an estimate of the fraction of the transcriptome covered by the number of cells we have in our scRNA-seq dataset.

### Identification and evaluation of housekeeping genes

To calculate the skewness score, we computed the percentage of cells within every cell type expressing each gene present in our scRNA-seq data. We then used baseR’s skewness function to score the skewness of every gene with respect to their percent of cells expressed within each cell type: a negative value (left skew) indicating expression in the majority of cells and cell types and a positive value (right skew) indicating expression in the minority of cells and cell types. To compute the Gc for every gene across cell types, we used the ineq function from the ineq package on the scaled TPM gene by cell type matrix. To perform Gene Ontology enrichment analysis, we used Wormbase’s gene set enrichment analysis tool with the default *q* value threshold of 0.1. To measure the enrichment of essential genes in our various housekeeping genes list, we first downloaded the list of genes annotated as “embryonic lethal,” “larval lethal,” and “adult lethal” from Wormbase. We combined these lists into a final list of essential genes made up of 3275 genes. We then used Fisher’s exact test to determine the extent of enrichment of essential genes in our housekeeping genes lists.

### Inferring transcriptional regulators underlying cellular identity

To infer the potential role of TFs in mediating cell type–specific gene expression, we correlated TF binding patterns obtained from ChIP-Seq analysis with the gene expression profile of each cell type. We first collected all the available ChIP-Seq data from the modENCODE/modERN projects (*23*–*25*). All ChIP-Seq data are currently available from the ENCODE Data Coordination Center. We included in the analysis ChIP-Seq data performed in any post-embryonic stage (276 TFs) and ChIP-Seq data at embryonic stages (87 TFs) if the TF had not been tested post-embryonically. The ChIP-Seq peaks were then clustered along the genome by sorting the peaks by the apex base position of the peak. The peaks were accumulated into clusters moving along the genome until a gap of 200 bases between peaks was encountered, at which point a new cluster was begun. This resulted in 56,729 clusters, varying in size between 1 TF and hundreds of TFs. Clusters that contained more than 70 TFs were excluded from the analysis because these are considered HOT (high occupancy target) sites and are not likely to represent tissue-specific binding events. Similarly, clusters containing a single TF were also excluded because they are likely enriched in spurious binding. The target genes of the peak clusters were assigned by proximity of the cluster to the transcription start site (TSS) of the nearby genes. If the average of the apex of the peaks in the cluster met two criteria, then the cluster was assigned to the gene with the closest TSS. The first criterion was that the peak cluster must be within 2000 bases of the nearest gene TSS. The second criterion was that the distance to the next closest gene TSS must be at least 1.5 times the distance to the nearest gene TSS. The peaks in each experiment (TF/stage) were ranked by signal strength and normalized to a cumulative probability. We then used a matrix containing the normalized signal strength as values, the TF as columns, and the target genes as rows as the predictor variable matrix input for a generalized linear model (glmnet in R). If the cluster had multiple peaks of a given TF or there were multiple clusters assigned to the same target with the same TF, then the maximum signal strength for the TF was used in the predictor variable matrix. The response vector for the model was the aggregated gene by cell type matrix we generated from our scRNA-seq data. We then ran a separate model for each cell type, generating a determined coefficient for each TF cell type association. These coefficients were used to generate the heatmaps found in Fig. 3 (D to F) (data S4). Any negative coefficients were set to 0 in the heatmap. Last, we performed a 20-fold cross-validation to determine the mean square error for the cell type model. That number was appended to each cell type (data S4) with a lower number indicating a higher confidence in the predictions of the model.

### Inferring cell-cell communication from the gene expression of ligands and receptors in cells

To study CCIs, we used a list of 245 LR interactions of *C. elegans* (*35*). We used *cell2cell* by using the pipeline cell2cell.analysis.SingleCellExperiment found in the *cell2cell* python package, which allows running a permutation analysis for computing the significance of the inferred communication scores for each combination of LR interaction and sender-receiver cell pairs, as previously introduced (*36*). To run this analysis, the expression level of each gene was aggregated at the cell type level by computing the log1p(counts per million or CPM) average expression within each cluster. Then, the communication score was computed as the geometric mean of the expression of the ligand in a sender cell type and the receptor in a receiver cell type.

To run *Tensor-cell2cell* to identify latent patterns of communication, only the communication scores with a *P* value < 0.05 (indicating cell-type specific CCI) were used to build a 3D communication tensor (fig. S5A). This 3D tensor was decomposed by using *Tensor-cell2cell* into seven factors, each factor representing a signature or module of CCI that summarizes a biological process involving specific cell types and LR pairs.

### Levamisole treatment

The levamisole assays were performed as previously described (*72*) with some modifications. N2 and tissue-specific RNAi *C. elegans* strains were bleached, and the embryos were rocked at 20°C for 18 hours to synchronize the hatchlings. We then seeded around 200 hatchlings on NGM + 1 mM isopropyl-β-d-thiogalactopyranoside + carbenicillin plates (25 μg/ml; RNAi plates) seeded with *E. coli* strain HT115 carrying an empty L4440 plasmid (control). A day before the worms became L4s, HT115 carrying either an empty plasmid or an L4440 plasmid carrying the gene of interest was seeded on 24-well RNAi plates. Once the worms reached the L4 stage, we moved ~20 to 40 worms to each bacteria-seeded well. After 24 hours, the wells were flushed in a sequential manner with 1 ml of 0.4 mM levamisole. Every 10 min, we counted the number of moving worms until all worms were paralyzed. Results were analyzed on SPSS using the Kaplan-Meier estimate with log rank test comparison across different strata. Figures were made using GraphPad Prism.

### Statistical analysis

All statistical analyses were performed in R except for the levamisole analysis which was analyzed in SPSS. All statistical analyses are described in their appropriate section in the main text, figure legend and methods. We used Fisher’s exact test to perform enrichment analyses (the lower limit of the *P* value in R is 2.2 × 10^−16^). Correlation analysis between different datasets was performed using Pearson correlation coefficient, and correlation analysis between different dendrograms was performed using Baker’s gamma correlation coefficient available through the dendextend R package (*73*). The levamisole data was analyzed using the Kaplan-Meier estimator with log-rank test comparison across different strata. We considered *P* values < 0.05 as statistically significant.

## References

[1] J. S. Packer, Q. Zhu, C. Huynh, P. Sivaramakrishnan, E. Preston, H. Dueck, D. Stefanik, K. Tan, C. Trapnell, J. Kim, R. H. Waterston, J. I. Murray, A lineage-resolved molecular atlas of *C. elegans* embryogenesis at single-cell resolution. Science 365, eaax1971 (2019). 31488706 10.1126/science.aax1971 PMC7428862

[2] J. E. Sulston, H. R. Horvitz, Post-embryonic cell lineages of the nematode, Caenorhabditis elegans. Dev. Biol. 56, 110–156 (1977). 838129 10.1016/0012-1606(77)90158-0

[3] S. R. Taylor, G. Santpere, A. Weinreb, A. Barrett, M. B. Reilly, C. Xu, E. Varol, P. Oikonomou, L. Glenwinkel, R. McWhirter, A. Poff, M. Basavaraju, I. Rafi, E. Yemini, S. J. Cook, A. Abrams, B. Vidal, C. Cros, S. Tavazoie, N. Sestan, M. Hammarlund, O. Hobert, D. M. Miller, Molecular topography of an entire nervous system. Cell 184, 4329–4347.e23 (2021). 34237253 10.1016/j.cell.2021.06.023 PMC8710130

[4] R. Kaletsky, V. Yao, A. Williams, A. M. Runnels, A. Tadych, S. Zhou, O. G. Troyanskaya, C. T. Murphy, Transcriptome analysis of adult Caenorhabditis elegans cells reveals tissue-specific gene and isoform expression. PLOS Genet. 14, e1007559 (2018). 30096138 10.1371/journal.pgen.1007559 PMC6105014

[5] W. C. Spencer, R. McWhirter, T. Miller, P. Strasbourger, O. Thompson, L. W. Hillier, R. H. Waterston, D. M. Miller, Isolation of specific neurons from C. elegans larvae for gene expression profiling. PLOS ONE 9, e112102 (2014). 25372608 10.1371/journal.pone.0112102 PMC4221280

[6] J. Cao, J. S. Packer, V. Ramani, D. A. Cusanovich, C. Huynh, R. Daza, X. Qiu, C. Lee, S. N. Furlan, F. J. Steemers, A. Adey, R. H. Waterston, C. Trapnell, J. Shendure, Comprehensive single-cell transcriptional profiling of a multicellular organism. Science 357, 661–667 (2017). 28818938 10.1126/science.aam8940 PMC5894354

[7] S. C. Tintori, E. O. Nishimura, P. Golden, J. D. Lieb, B. Goldstein, A transcriptional lineage of the early C. elegans embryo. Dev. Cell 38, 430–444 (2016). 27554860 10.1016/j.devcel.2016.07.025 PMC4999266

[8] A. E. Roux, H. Yuan, K. Podshivalova, D. Hendrickson, R. Kerr, C. Kenyon, D. R. Kelley, The complete cell atlas of an aging multicellular organism. bioRxiv, 2022.06.15.496201 (2022).

[9] J. Cao, M. Spielmann, X. Qiu, X. Huang, D. M. Ibrahim, A. J. Hill, F. Zhang, S. Mundlos, L. Christiansen, F. J. Steemers, C. Trapnell, J. Shendure, The single-cell transcriptional landscape of mammalian organogenesis. Nature 566, 496–502 (2019). 30787437 10.1038/s41586-019-0969-x PMC6434952

[10] P. Davis, M. Zarowiecki, V. Arnaboldi, A. Becerra, S. Cain, J. Chan, W. J. Chen, J. Cho, E. da Veiga Beltrame, S. Diamantakis, S. Gao, D. Grigoriadis, C. A. Grove, T. W. Harris, R. Kishore, T. Le, R. Y. N. Lee, M. Luypaert, H.-M. Müller, C. Nakamura, P. Nuin, M. Paulini, M. Quinton-Tulloch, D. Raciti, F. H. Rodgers, M. Russell, G. Schindelman, A. Singh, T. Stickland, K. Van Auken, Q. Wang, G. Williams, A. J. Wright, K. Yook, M. Berriman, K. L. Howe, T. Schedl, L. Stein, P. W. Sternberg, WormBase in 2022—Data, processes, and tools for analyzing *Caenorhabditis elegans*. Genetics 220, iyac003 (2022). 35134929 10.1093/genetics/iyac003 PMC8982018

[11] O. Hobert, L. Glenwinkel, J. White, Revisiting neuronal cell type classification in Caenorhabditis elegans. Curr. Biol. 26, R1197–R1203 (2016). 27875702 10.1016/j.cub.2016.10.027

[12] C. R. Gissendanner, K. Kelley, T. Q. Nguyen, M. C. Hoener, A. E. Sluder, C. V. Maina, The Caenorhabditis elegans NR4A nuclear receptor is required for spermatheca morphogenesis. Dev. Biol. 313, 767–786 (2008). 18096150 10.1016/j.ydbio.2007.11.014 PMC3845373

[13] S. Nadarajan, J. A. Govindan, M. McGovern, E. J. A. Hubbard, D. Greenstein, MSP and GLP-1/Notch signaling coordinately regulate actomyosin-dependent cytoplasmic streaming and oocyte growth in *C. elegans*. Development 136, 2223–2234 (2009). 19502484 10.1242/dev.034603 PMC2729341

[14] S. Aono, R. Legouis, W. A. Hoose, K. J. Kemphues, PAR-3 is required for epithelial cell polarity in the distal spermatheca of C. elegans. Development 131, 2865–2874 (2004). 15151982 10.1242/dev.01146

[15] S. Ward, J. S. Carrel, Fertilization and sperm competition in the nematode Caenorhabditis elegans. Dev. Biol. 73, 304–321 (1979). 499670 10.1016/0012-1606(79)90069-1

[16] J. Hodgkin, T. Doniach, Natural variation and copulatory plug formation in Caenorhabditis elegans. Genetics 146, 149–164 (1997). 9136008 10.1093/genetics/146.1.149 PMC1207933

[17] C. J. Joshi, W. Ke, A. Drangowska-Way, E. J. O’Rourke, N. E. Lewis, What are housekeeping genes? PLOS Comput. Biol. 18, e1010295 (2022). 35830477 10.1371/journal.pcbi.1010295 PMC9312424

[18] R. S. Kamath, A. G. Fraser, Y. Dong, G. Poulin, R. Durbin, M. Gotta, A. Kanapin, N. Le Bot, S. Moreno, M. Sohrmann, D. P. Welchman, P. Zipperlen, J. Ahringer, Systematic functional analysis of the Caenorhabditis elegans genome using RNAi. Nature 421, 231–237 (2003). 12529635 10.1038/nature01278

[19] F. Simmer, C. Moorman, A. M. van der Linden, E. Kuijk, P. V. E. van den Berghe, R. S. Kamath, A. G. Fraser, J. Ahringer, R. H. A. Plasterk, Genome-wide RNAi of C. elegans using the hypersensitive rrf-3 strain reveals novel gene functions. PLOS Biol. 1, E12 (2003). 14551910 10.1371/journal.pbio.0000012 PMC212692

[20] Y. Tabach, A. C. Billi, G. D. Hayes, M. A. Newman, O. Zuk, H. Gabel, R. Kamath, K. Yacoby, B. Chapman, S. M. Garcia, M. Borowsky, J. K. Kim, G. Ruvkun, Identification of small RNA pathway genes using patterns of phylogenetic conservation and divergence. Nature 493, 694–698 (2013). 23364702 10.1038/nature11779 PMC3762460

[21] Y. Zhang, D. Chen, M. A. Smith, B. Zhang, X. Pan, Selection of reliable reference genes in Caenorhabditis elegans for analysis of nanotoxicity. PLOS ONE 7, e31849 (2012). 22438870 10.1371/journal.pone.0031849 PMC3305280

[22] J. Tao, Y. Hao, X. Li, H. Yin, X. Nie, J. Zhang, B. Xu, Q. Chen, B. Li, Systematic identification of housekeeping genes possibly used as references in Caenorhabditis elegans by large-scale data integration. Cell 9, 786 (2020). 10.3390/cells9030786 PMC7140892 32213971

[23] C. L. Araya, T. Kawli, A. Kundaje, L. Jiang, B. Wu, D. Vafeados, R. Terrell, P. Weissdepp, L. Gevirtzman, D. Mace, W. Niu, A. P. Boyle, D. Xie, L. Ma, J. I. Murray, V. Reinke, R. H. Waterston, M. Snyder, Corrigendum: Regulatory analysis of the C. elegans genome with spatiotemporal resolution. Nature 528, 152 (2015). 10.1038/nature16075 26560031

[24] An Integrated encyclopedia of DNA elements in the human genome. Nature 489, 57–74 (2012). 22955616 10.1038/nature11247 PMC3439153

[25] M. M. Kudron, A. Victorsen, L. Gevirtzman, L. W. Hillier, W. W. Fisher, D. Vafeados, M. Kirkey, A. S. Hammonds, J. Gersch, H. Ammouri, M. L. Wall, J. Moran, D. Steffen, M. Szynkarek, S. Seabrook-Sturgis, N. Jameel, M. Kadaba, J. Patton, R. Terrell, M. Corson, T. J. Durham, S. Park, S. Samanta, M. Han, J. Xu, K.-K. Yan, S. E. Celniker, K. P. White, L. Ma, M. Gerstein, V. Reinke, R. H. Waterston, The ModERN resource: Genome-wide binding profiles for hundreds of Drosophila and Caenorhabditis elegans transcription factors. Genetics 208, 937–949 (2018). 29284660 10.1534/genetics.117.300657 PMC5844342

[26] T. Fukushige, T. M. Brodigan, L. A. Schriefer, R. H. Waterston, M. Krause, Defining the transcriptional redundancy of early bodywall muscle development in C. elegans: Evidence for a unified theory of animal muscle development. Genes Dev. 20, 3395–3406 (2006). 17142668 10.1101/gad.1481706 PMC1698447

[27] C. R. Gissendanner, A. E. Sluder, nhr-25, the Caenorhabditis elegans ortholog of ftz-f1, is required for epidermal and somatic gonad development. Dev. Biol. 221, 259–272 (2000). 10772806 10.1006/dbio.2000.9679

[28] J. S. Gilleard, J. D. McGhee, Activation of hypodermal differentiation in the Caenorhabditis elegans embryo by GATA transcription factors ELT-1 and ELT-3. Mol. Cell. Biol. 21, 2533–2544 (2001). 11259601 10.1128/MCB.21.7.2533-2544.2001 PMC86885

[29] C. Brabin, P. J. Appleford, A. Woollard, The Caenorhabditis elegans GATA factor ELT-1 works through the cell proliferation regulator BRO-1 and the Fusogen EFF-1 to maintain the seam stem-like fate. PLOS Genet. 7, e1002200 (2011). 21829390 10.1371/journal.pgen.1002200 PMC3150447

[30] T. Fukushige, M. G. Hawkins, J. D. McGhee, The GATA-factor elt-2 is essential for formation of the Caenorhabditis elegans intestine. Dev. Biol. 198, 286–302 (1998). 9659934

[31] R. G. Tepper, J. Ashraf, R. Kaletsky, G. Kleemann, C. T. Murphy, H. J. Bussemaker, PQM-1 complements DAF-16 as a key transcriptional regulator of DAF-2-mediated development and longevity. Cell 154, 676–690 (2013). 23911329 10.1016/j.cell.2013.07.006 PMC3763726

[32] J. Gaudet, S. E. Mango, Regulation of organogenesis by the Caenorhabditis elegans FoxA protein PHA-4. Science 295, 821–825 (2002). 11823633 10.1126/science.1065175

[33] O. Hobert, A map of terminal regulators of neuronal identity in Caenorhabditis elegans. WIREs Dev. Biol. 5, 474–498 (2016). 10.1002/wdev.233 PMC4911249 27136279

[34] R. Branicky, S. Hekimi, Specification of muscle neurotransmitter sensitivity by a Paired-like homeodomain protein in *Caenorhabditis elegans*. Development 132, 4999–5009 (2005). 16236771 10.1242/dev.02069

[35] E. Armingol, A. Ghaddar, C. J. Joshi, H. Baghdassarian, I. Shamie, J. Chan, H.-L. Her, S. Berhanu, A. Dar, F. Rodriguez-Armstrong, O. Yang, E. J. O’Rourke, N. E. Lewis, Inferring a spatial code of cell-cell interactions across a whole animal body. PLOS Comput. Biol. 18, e1010715 (2022). 36395331 10.1371/journal.pcbi.1010715 PMC9714814

[36] M. Efremova, M. Vento-Tormo, S. A. Teichmann, R. Vento-Tormo, CellPhoneDB: Inferring cell–cell communication from combined expression of multi-subunit ligand–receptor complexes. Nat. Protoc. 15, 1484–1506 (2020). 32103204 10.1038/s41596-020-0292-x

[37] S. Jin, C. F. Guerrero-Juarez, L. Zhang, I. Chang, R. Ramos, C.-H. Kuan, P. Myung, M. V. Plikus, Q. Nie, Inference and analysis of cell-cell communication using CellChat. Nat. Commun. 12, 1088 (2021). 33597522 10.1038/s41467-021-21246-9 PMC7889871

[38] S. T. Henderson, D. Gao, E. J. Lambie, J. Kimble, lag-2 may encode a signaling ligand for the GLP-1 and LIN-12 receptors of C. elegans. Development 120, 2913–2924 (1994). 7607081 10.1242/dev.120.10.2913

[39] M. Chalfie, J. E. Sulston, J. G. White, E. Southgate, J. N. Thomson, S. Brenner, The neural circuit for touch sensitivity in Caenorhabditis elegans. J. Neurosci. 5, 956–964 (1985). 3981252 10.1523/JNEUROSCI.05-04-00956.1985 PMC6565008

[40] F. Calahorro, M. Ruiz-Rubio, Functional phenotypic rescue of Caenorhabditis elegans neuroligin-deficient mutants by the human and rat NLGN1 genes. PLOS ONE 7, e39277 (2012). 22723984 10.1371/journal.pone.0039277 PMC3377638

[41] X. Wang, J. Kweon, S. Larson, L. Chen, A role for the C. elegans L1CAM homologue lad-1/sax-7 in maintaining tissue attachment. Dev. Biol. 284, 273–291 (2005). 16023097 10.1016/j.ydbio.2005.05.020

[42] Z. Chen, M. Hendricks, A. Cornils, W. Maier, J. Alcedo, Y. Zhang, Two insulin-like peptides antagonistically regulate aversive olfactory learning in C. elegans. Neuron 77, 572–585 (2013). 23395381 10.1016/j.neuron.2012.11.025 PMC3569836

[43] A. Cornils, M. Gloeck, Z. Chen, Y. Zhang, J. Alcedo, Specific insulin-like peptides encode sensory information to regulate distinct developmental processes. Development 138, 1183–1193 (2011). 21343369 10.1242/dev.060905 PMC3042873

[44] C. T. Murphy, S.-J. Lee, C. Kenyon, Tissue entrainment by feedback regulation of insulin gene expression in the endoderm of Caenorhabditis elegans. Proc. Natl. Acad. Sci. U.S.A. 104, 19046–19050 (2007). 18025456 10.1073/pnas.0709613104 PMC2141905

[45] W. Li, S. G. Kennedy, G. Ruvkun, *daf-28* encodes a *C. elegans* insulin superfamily member that is regulated by environmental cues and acts in the DAF-2 signaling pathway. Genes Dev. 17, 844–858 (2003). 12654727 10.1101/gad.1066503 PMC196030

[46] S. B. Pierce, M. Costa, R. Wisotzkey, S. Devadhar, S. A. Homburger, A. R. Buchman, K. C. Ferguson, J. Heller, D. M. Platt, A. A. Pasquinelli, L. X. Liu, S. K. Doberstein, G. Ruvkun, Regulation of DAF-2 receptor signaling by human insulin and ins-1, a member of the unusually large and diverse C. elegans insulin gene family. Genes Dev. 15, 672–686 (2001). 11274053 10.1101/gad.867301 PMC312654

[47] D. Michaelson, D. Z. Korta, Y. Capua, E. J. A. Hubbard, Insulin signaling promotes germline proliferation in *C. elegans*. Development 137, 671–680 (2010). 20110332 10.1242/dev.042523 PMC2827619

[48] W. L. Hung, Y. Wang, J. Chitturi, M. Zhen, A *Caenorhabditis elegans* developmental decision requires insulin signaling-mediated neuron-intestine communication. Development 141, 1767–1779 (2014). 24671950 10.1242/dev.103846 PMC3978837

[49] M. Tomioka, T. Adachi, H. Suzuki, H. Kunitomo, W. R. Schafer, Y. Iino, The insulin/PI 3-kinase pathway regulates salt chemotaxis learning in Caenorhabditis elegans. Neuron 51, 613–625 (2006). 16950159 10.1016/j.neuron.2006.07.024

[50] B. D. Ackley, S. H. Kang, J. R. Crew, C. Suh, Y. Jin, J. M. Kramer, The basement membrane components nidogen and type XVIII collagen regulate organization of neuromuscular junctions in *Caenorhabditis elegans*. J. Neurosci. 23, 3577–3587 (2003). 12736328 10.1523/JNEUROSCI.23-09-03577.2003 PMC6742194

[51] B. D. Williams, R. H. Waterston, Genes critical for muscle development and function in Caenorhabditis elegans identified through lethal mutations. J. Cell Biol. 124, 475–490 (1994). 8106547 10.1083/jcb.124.4.475 PMC2119919

[52] T. Hikita, H. Qadota, D. Tsuboi, S. Taya, D. G. Moerman, K. Kaibuchi, Identification of a novel Cdc42 GEF that is localized to the PAT-3-mediated adhesive structure. Biochem. Biophys. Res. Commun. 335, 139–145 (2005). 16055082 10.1016/j.bbrc.2005.07.068

[53] J. A. Lewis, C.-H. Wu, H. Berg, J. H. Levine, The genetics of levamisole resistance in the nematode *Caenorhabditis elegans*. Genetics 95, 905–928 (1980). 7203008 10.1093/genetics/95.4.905 PMC1214276

[54] J. A. Lewis, C. H. Wu, J. H. Levine, H. Berg, Levamisole-resistant mutants of the nematode Caenorhabditis elegans appear to lack pharmacological acetylcholine receptors. Neuroscience 5, 967–989 (1980). 7402460 10.1016/0306-4522(80)90180-3

[55] H. Qian, A. P. Robertson, J. A. Powell-Coffman, R. J. Martin, Levamisole resistance resolved at the single-channel level in Caenorhabditis elegans. FASEB J. 22, 3247–3254 (2008). 18519804 10.1096/fj.08-110502 PMC2518249

[56] A. Calixto, D. Chelur, I. Topalidou, X. Chen, M. Chalfie, Enhanced neuronal RNAi in C. elegans using SID-1. Nat. Methods 7, 554–559 (2010). 20512143 10.1038/nmeth.1463 PMC2894993

[57] Tabula Muris Consortium; Overall coordination; Logistical coordination; Organ collection and processing; Library preparation and sequencing; Computational data analysis; Cell type annotation; Writing group; Supplemental text writing group; Principal investigators, Single-cell transcriptomics of 20 mouse organs creates a Tabula Muris. Nature 562, 367–372 (2018). 30283141 10.1038/s41586-018-0590-4 PMC6642641

[58] Tabula Sapiens Consortium, The Tabula Sapiens: A multiple-organ, single-cell transcriptomic atlas of humans. Science 376, eabl4896 (2022). 35549404 10.1126/science.abl4896 PMC9812260

[59] D. Grün, M. Kirchner, N. Thierfelder, M. Stoeckius, M. Selbach, N. Rajewsky, Conservation of mRNA and protein expression during development of C. elegans. Cell Rep. 6, 565–577 (2014). 24462290 10.1016/j.celrep.2014.01.001

[60] D. M. Walther, P. Kasturi, M. Zheng, S. Pinkert, G. Vecchi, P. Ciryam, R. I. Morimoto, C. M. Dobson, M. Vendruscolo, M. Mann, F. U. Hartl, Widespread proteome remodeling and aggregation in aging C. elegans. Cell 161, 919–932 (2015). 25957690 10.1016/j.cell.2015.03.032 PMC4643853

[61] E. B. Harvald, R. R. Sprenger, K. B. Dall, C. S. Ejsing, R. Nielsen, S. Mandrup, A. B. Murillo, M. Larance, A. Gartner, A. I. Lamond, N. J. Færgeman, Multi-omics analyses of starvation responses reveal a central role for lipoprotein metabolism in acute starvation survival in C. elegans. Cell Syst. 5, 38–52.e4 (2017). 28734827 10.1016/j.cels.2017.06.004

[62] M. McGovern, R. Voutev, J. Maciejowski, A. K. Corsi, E. J. A. Hubbard, A “latent niche” mechanism for tumor initiation. Proc. Natl. Acad. Sci. U.S.A. 106, 11617–11622 (2009). 19564624 10.1073/pnas.0903768106 PMC2710656

[63] R. A. Green, H.-L. Kao, A. Audhya, S. Arur, J. R. Mayers, H. N. Fridolfsson, M. Schulman, S. Schloissnig, S. Niessen, K. Laband, S. Wang, D. A. Starr, A. A. Hyman, T. Schedl, A. Desai, F. Piano, K. C. Gunsalus, K. Oegema, A high-resolution C. elegans essential gene network based on phenotypic profiling of a complex tissue. Cell 145, 470–482 (2011). 21529718 10.1016/j.cell.2011.03.037 PMC3086541

[64] J. S. Reece-Hoyes, B. Deplancke, J. Shingles, C. A. Grove, I. A. Hope, A. J. Walhout, A compendium of Caenorhabditis elegans regulatory transcription factors: A resource for mapping transcription regulatory networks. Genome Biol. 6, R110 (2005). 16420670 10.1186/gb-2005-6-13-r110 PMC1414109

[65] E. Armingol, H. M. Baghdassarian, C. Martino, A. Perez-Lopez, C. Aamodt, R. Knight, N. E. Lewis, Context-aware deconvolution of cell–cell communication with Tensor-cell2cell. Nat. Commun. 13, 3665 (2022). 35760817 10.1038/s41467-022-31369-2 PMC9237099

[66] F. Long, H. Peng, X. Liu, S. K. Kim, E. Myers, A 3D digital atlas of C. elegans and its application to single-cell analyses. Nat. Methods 6, 667–672 (2009). 19684595 10.1038/nmeth.1366 PMC2882208

[67] A. G. Fraser, R. S. Kamath, P. Zipperlen, M. Martinez-Campos, M. Sohrmann, J. Ahringer, Functional genomic analysis of C. elegans chromosome I by systematic RNA interference. Nature 408, 325–330 (2000). 11099033 10.1038/35042517

[68] S. Zhang, D. Banerjee, J. R. Kuhn, Isolation and culture of larval cells from *C. elegans*. PLOS ONE 6, e19505 (2011). 21559335 10.1371/journal.pone.0019505 PMC3084877

[69] S. Yang, S. E. Corbett, Y. Koga, Z. Wang, W. E. Johnson, M. Yajima, J. D. Campbell, Decontamination of ambient RNA in single-cell RNA-seq with DecontX. Genome Biol. 21, 57 (2020). 32138770 10.1186/s13059-020-1950-6 PMC7059395

[70] T. Ilicic, J. K. Kim, A. A. Kolodziejczyk, F. O. Bagger, D. J. McCarthy, J. C. Marioni, S. A. Teichmann, Classification of low quality cells from single-cell RNA-seq data. Genome Biol. 17, 29 (2016). 26887813 10.1186/s13059-016-0888-1 PMC4758103

[71] C. Ritz, F. Baty, J. C. Streibig, D. Gerhard, Dose-response analysis using R. PLOS ONE 10, e0146021 (2015). 26717316 10.1371/journal.pone.0146021 PMC4696819

[72] A. N. Davis, J. E. Tanis, Measuring *Caenorhabditis elegans* sensitivity to the acetylcholine receptor agonist levamisole. J. Vis. Exp., e64056 (2022). 10.3791/64056 PMC10016203 35758705

[73] T. Galili, dendextend: An R package for visualizing, adjusting and comparing trees of hierarchical clustering. Bioinformatics 31, 3718–3720 (2015). 26209431 10.1093/bioinformatics/btv428 PMC4817050

[74] F. Murtagh, P. Legendre, Ward’s hierarchical agglomerative clustering method: Which algorithms implement ward’s criterion? J. Classif. 31, 274–295 (2014).

