# Supplementary material for: Whole-body gene expression atlas of an adult metazoan
Source: Sci Adv. 2023 Jun 23;9(25):eadg0506. doi: 10.1126/sciadv.adg0506 (PMC10289653; doi:10.1126/sciadv.adg0506)
Supplement: Supplementary file 1 — Figs. S1 to S5 Legends for data S1 to S7 [file sciadv.adg0506_sm.pdf]

Science Advances
AAAS

Supplementary Materials for

Whole-body gene expression atlas of an adult metazoan

Abbas Ghaddar *et al.*

Corresponding author: Eyleen J. O'Rourke, ejorourke@virginia.edu

Sci. Adv. **9**, eadg0506 (2023)
DOI: 10.1126/sciadv.adg0506

The PDF file includes:

Figs. S1 to S5
Legends for data S1 to S7

Other Supplementary Material for this manuscript includes the following:

Data S1 to S7

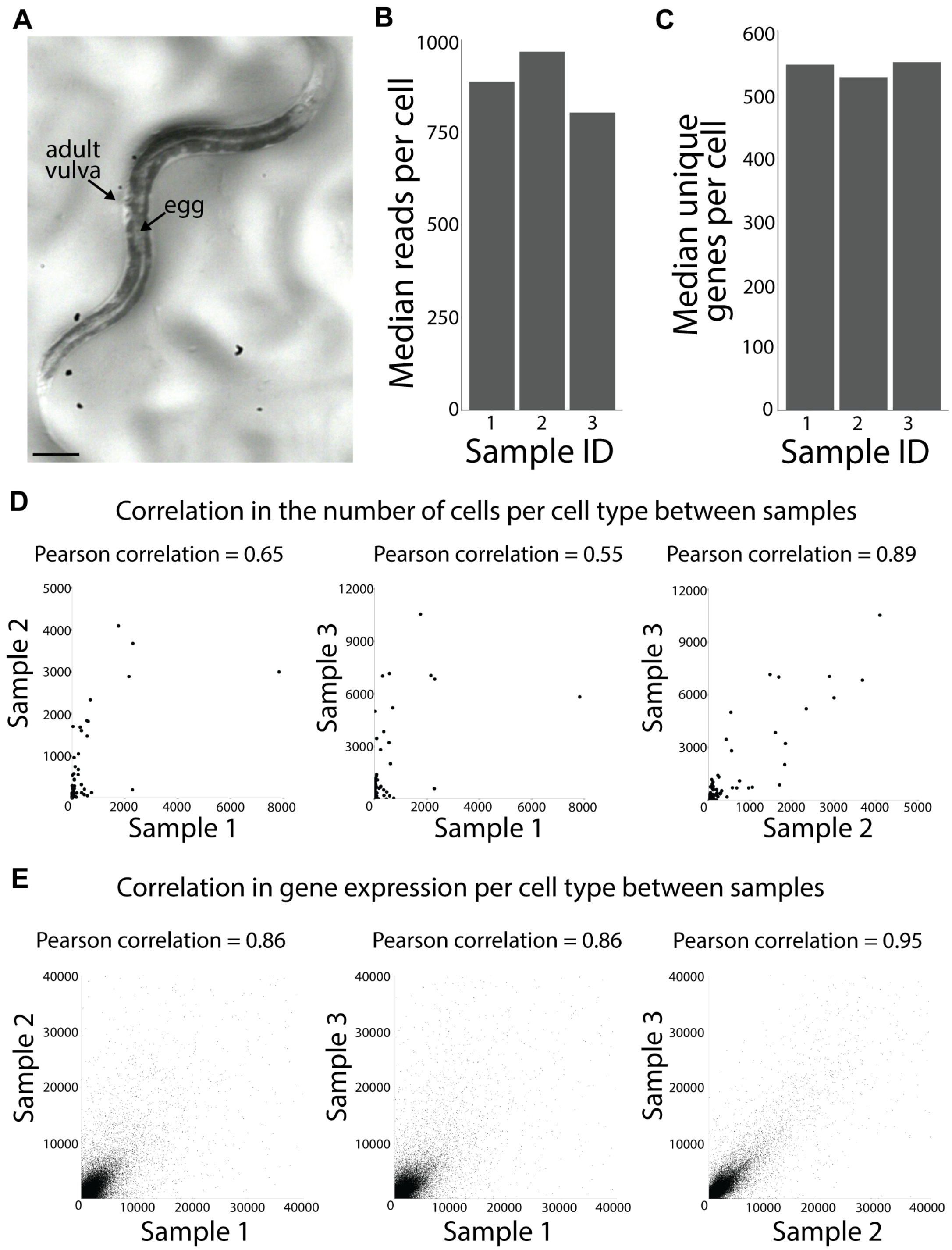

Fig. S1. scRNA-Seq of young adult *C. elegans* **(A)** Representative image of young adult *C. elegans,* which we identified by the characteristic shape of the vulva (arrow). Scale bar = 100 µm. **(B)** Average count per cell for each biological replicate. **(C)** Average gene per cell for each biological replicate. **(D)** Correlation in the number of cells per cell type between the three biological replicates. Each dot represents the number of cells in a particular cell type in two samples. **(E)** Correlation of the cell-type specific gene expression profiles between the three biological replicates. Each dot represents the levels of expression of a gene within a cell type (scaled TPM).

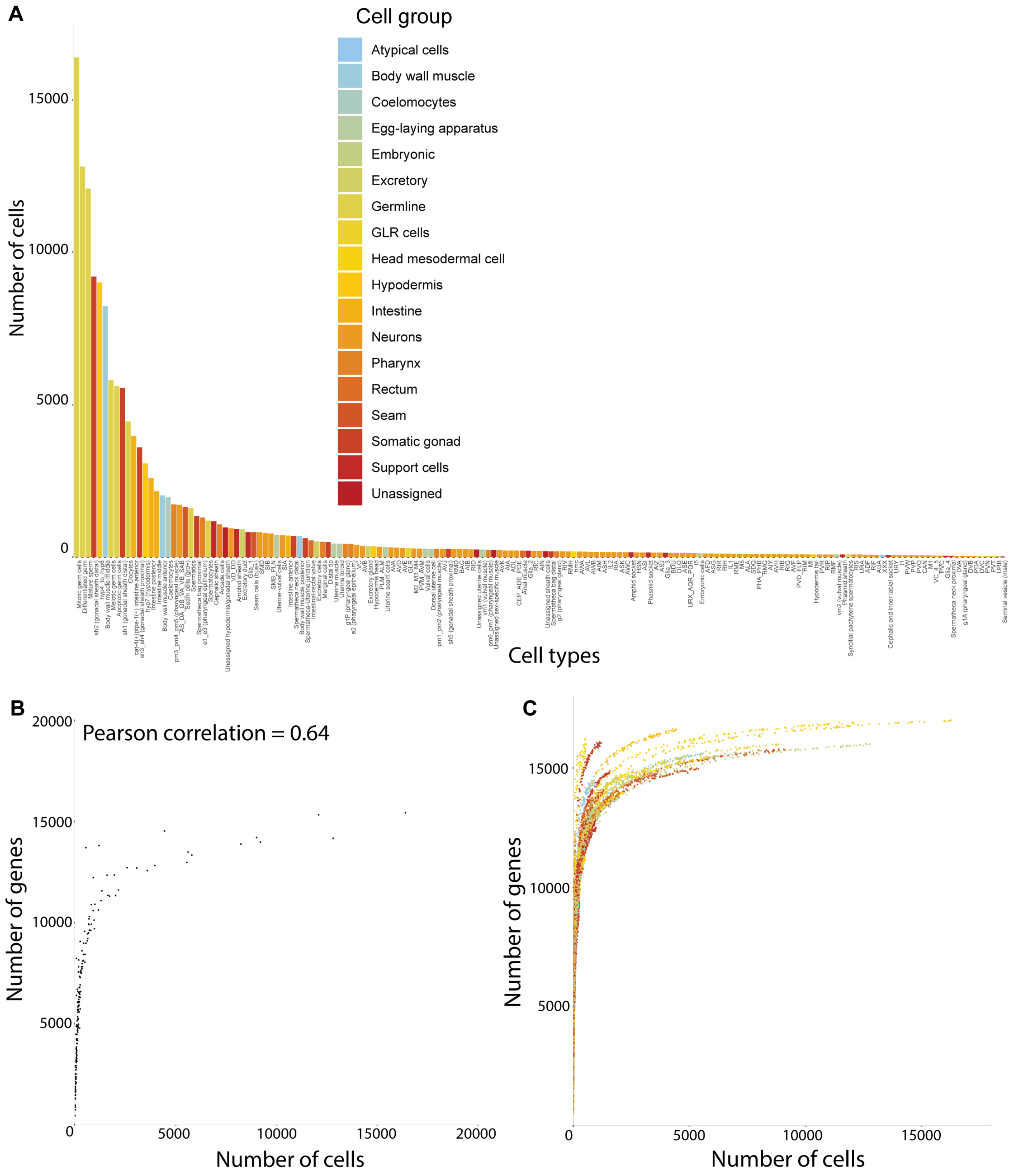

Fig. S2. Distribution of cell numbers per cell type and relationship between cell number and gene number. (A) Distribution of cell numbers per cell type. Subtypes of cells are colored by broadly defined cell types. **(B)** Number of genes detected for all cell types plotted against the number of cells. Pearson correlation coefficient = 0.64 shows a strong correlation between number of genes and number of cells. **(C)** Number of genes detected versus number of cells for all cell types after down sampling.

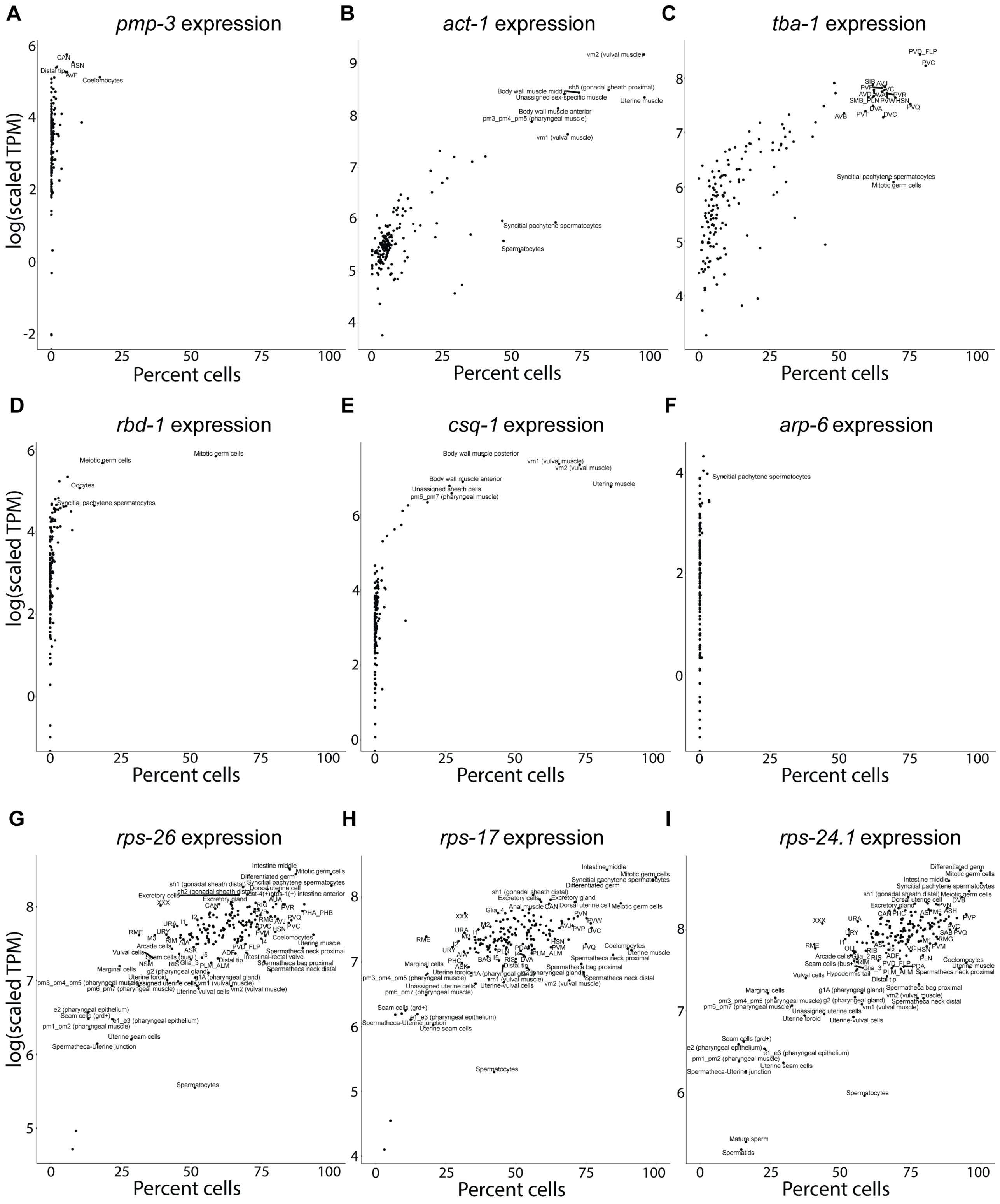

Fig. S3. Expression of commonly used housekeeping genes across cell types. (A-F) Gene expression of traditional housekeeping genes with high Gini coefficient (>0.5). Based on these results, these genes may not be appropriate housekeeping genes. **(G-I)** Gene expression of traditional housekeeping genes with low Gini coefficient (<0.3). These genes may be more appropriate housekeeping genes.

**Transcription factor**	**Cell type**
ceh-14	PVN
egl-13	BAG, URX, AQR, PQR
lim-6	DVB
lin-11	ADL, ASG, PVP
ttx-3	AIA
unc-3	SAB, DA, DB, VA, VB, AS, PDB, AVA, AVD, PDA
unc-42	ASH, AIB, AVA, AVD, AVE, AVK, SMD
unc-86	ALM, AQR, AVM, FLP, IL2, PLM, PQR, PVD, PVM, URA, URB, URX, AIM, AIZ, BDU, RIH, HSN

Fig. S4. List of TFs and the cell types in which they are known to mediate neuronal identity. These TF-cell type associations were confirmed by our TF analysis.

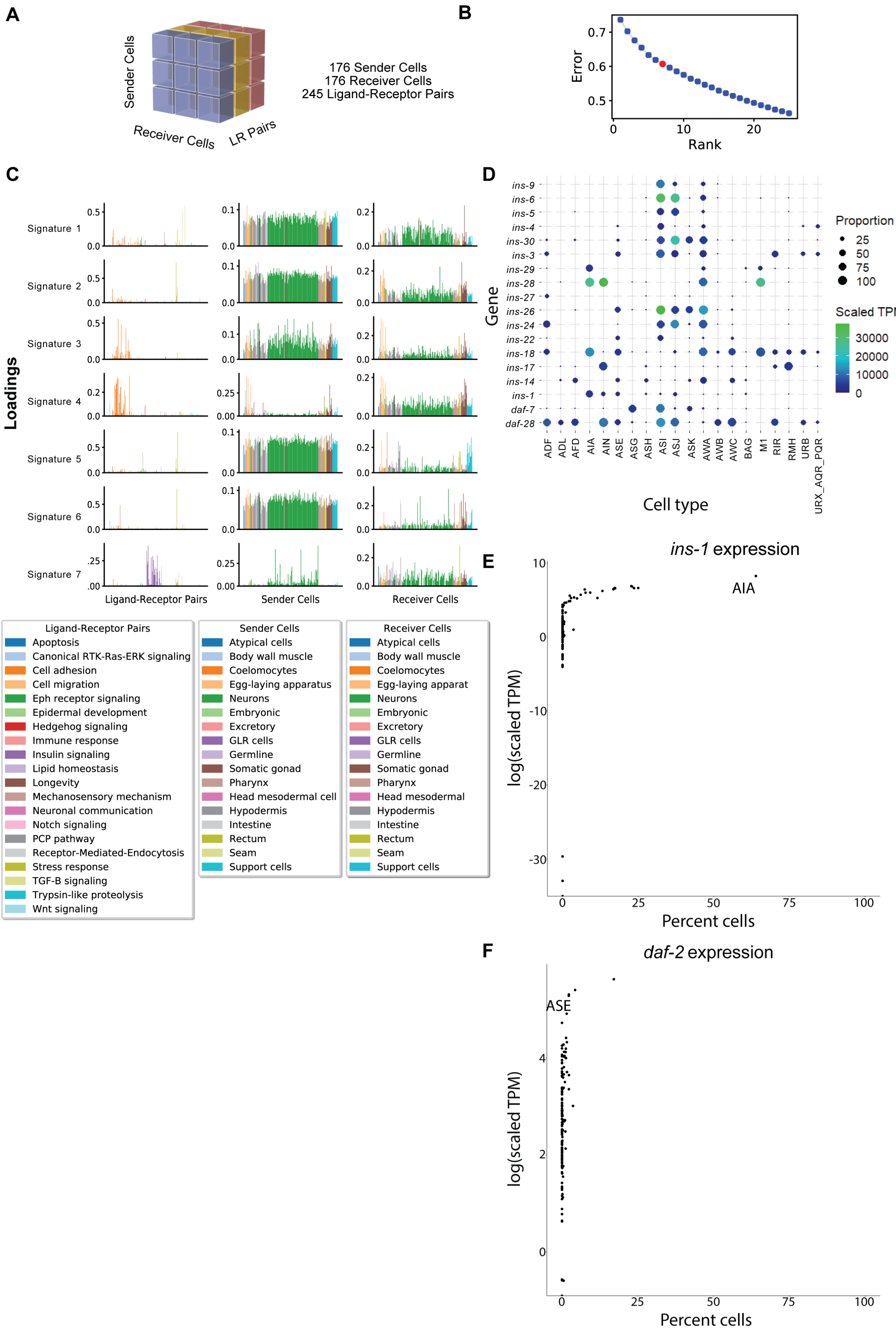

A
Sender Cells
Receiver Cells
LR Pairs
176 Sender Cells
176 Receiver Cells
245 Ligand-Receptor Pairs
B
Error
Rank
C
Loadings
Signature 1
Signature 2
Signature 3
Signature 4
Signature 5
Signature 6
Signature 7
Ligand-Receptor Pairs
Sender Cells
Receiver Cells
Ligand-Receptor Pairs
Apoptosis
Canonical RTK-Ras-ERK signaling
Cell adhesion
Cell migration
Eph receptor signaling
Epidermal development
Hedgehog signaling
Immune response
Insulin signaling
Lipid homeostasis
Longevity
Mechanosensory mechanism
Neuronal communication
Notch signaling
PCP pathway
Receptor-Mediated-Endocytosis
Stress response
TGF-B signaling
Trypsin-like proteolysis
Wnt signaling
Sender Cells
Atypical cells
Body wall muscle
Coelomocytes
Egg-laying apparatus
Neurons
Embryonic
Excretory
GLR cells
Germline
Somatic gonad
Pharynx
Head mesodermal cell
Hypodermis
Intestine
Rectum
Seam
Support cells
Receiver Cells
Atypical cells
Body wall muscle
Coelomocytes
Egg-laying apparat
Neurons
Embryonic
Excretory
GLR cells
Germline
Somatic gonad
Pharynx
Head mesodermal
Hypodermis
Intestine
Rectum
Seam
Support cells
D
Gene
ins-9
ins-6
ins-5
ins-4
ins-30
ins-3
ins-29
ins-28
ins-27
ins-26
ins-24
ins-22
ins-18
ins-17
ins-14
ins-1
daf-7
daf-28
ADF
ADL
AFD
AIA
AIN
ASE
ASG
ASH
ASI
ASJ
ASK
AWA
AWB
AWC
BAG
M1
RIR
RMH
URB
URX_AQR_PQR
Cell type
Proportion
25
50
75
100
Scaled TPM
30000
20000
10000
0
E
ins-1 expression
AIA
log(scaled TPM)
Percent cells
F
daf-2 expression
ASE
log(scaled TPM)
Percent cells

Fig. S5. Identification of cell-type specific communication signatures using Tensor-cell2cell. **(A)** Graphical representation of Tensor analysis. **(B)** Elbow plot to determine the optimal number of factors/signatures. **(C)** Tensor-cell2cell analysis: The first column of graphs represents the importance of each ligand-receptor in each signature colored by ligand-receptor pair class, the second column of graphs represents the importance of each sender cell in each signature colored by tissue type, the third column of graphs represents the importance of each receiver cell in each signature colored by tissue type. Color legend of functional, sender-cell, and receiver-cell classes can be found below the graphs. **(D)** The distribution of insulin ligands important across the sender cells in signature 7. **(E)** Enrichment of *ins-1* expression in AIA neurons. **(F)** Enrichment of *daf-2* expression in ASE neurons.

Data S1. (separate file)

This file includes the gene markers (output from Monocle3's find_markers function) and rationale used to annotate the various clusters as well as a list of the identified cell types. NOTE: some clusters contained several cell types. The cell types that could not be distinguished after sub-clustering are denoted by the "_". The cell types that could be distinguished after sub-clustering were annotated separately.

Data S2. (separate file)

This file includes the results of the analysis described in "Estimating transcriptome coverage for every cell type". Readers can find the actual number of cells identified and the actual number of genes identified per cell type in addition to the predicted maximum number of genes in every cell type (G_{MAX}) and the predicted number of cells required to identify half of G_{MAX}.

Data S3. (separate file)

This file contains several tables that pertain to the housekeeping genes analysis. **(A)** List of genes with negative skew. **(B)** List of genes with Gini < 0.3. **(C)** List of genes with Gini < 0.3 identified as housekeeping in L2. **(D)** List of genes with Gini < 0.3 identified as housekeeping in L2 and found to be essential in RNAi screens. **(E)** List of genes with Gini < 0.3 identified as housekeeping in L2, found to be essential in RNAi screens, and found to be conserved across species (Drosophila melanogaster, Danio rerio, Xenopus tropicalis, Gallus gallus, Homo sapiens, Mus musculus, Neurospora crassa, Saccharomyces cerevisiae, Arabidopsis thaliana).

Data S4. (separate file)

This file contains the results of the transcription factor analysis. The columns represent transcription factors, and the rows represent cell types. The values represent the correlation coefficient between the binding patterns of the transcription factors defined by ChIP-Seq and the gene expression profile of the cell types defined by scRNA-Seq. The numbers next to the cell types are the mean square error for the cell type model.

Data S5. (separate file)

This file includes the *dsc-1* targets expressed in the anal muscle that are known to play a role in **(A)** defecation/anal muscle function and, **(B)** muscle system morphology.

Data S6. (separate file)

This file includes the results of the Tensor-cell2cell analysis. **(A)** Loading values of ligand-receptor pairs per signature. **(B)** Loading values of sender cells per signature. **(C)** Loading values of receiver cells per signature.

Data S7. (separate file)

This file includes the raw and analyzed data of the levamisole sensitivity and NaCl chemotaxis assays. **(A)** Raw data of the levamisole sensitivity assay. **(B)** Analyzed data of the levamisole sensitivity assay. **(C)** Raw and analyzed data of the NaCl chemotaxis assay.
